# Acute care models for older people living with frailty: a systematic review and taxonomy

**DOI:** 10.1186/s12877-023-04373-4

**Published:** 2023-12-05

**Authors:** Thomas Knight, Vicky Kamwa, Catherine Atkin, Catherine Green, Janahan Ragunathan, Daniel Lasserson, Elizabeth Sapey

**Affiliations:** 1 Acute Care Research Group, Institute of Inflammation and Ageing, University of Birmingham, Birmingham, B15 2TT UK; 2 Department of Geriatric Medicine, Whiston Hospital, Mersey and West Lancashire Teaching Hospital NHS Trust, Prescot, L35 5DR UK; 3 Department of Geriatric Medicine, Royal Bolton NHS Foundation Trust, Bolton, BL4 0JR UK; 4 Warwick Medical School, Professor of Acute and Ambulatory Care, University of Warwick, Coventry, CV4 7AL UK

**Keywords:** Acute frailty, Care models, Taxonomy

## Abstract

**Background:**

The need to improve the acute care pathway to meet the care needs of older people living with frailty is a strategic priority for many healthcare systems. The optimal care model for this patient group is unclear.

**Methods:**

A systematic review was conducted to derive a taxonomy of acute care models for older people with acute medical illness and describe the outcomes used to assess their effectiveness. Care models providing time-limited episodes of care (up to 14 days) within 48 h of presentation to patients over the age of 65 with acute medical illness were included. Care models based in hospital and community settings were eligible.

Searches were undertaken in Medline, Embase, CINAHL and Cochrane databases. Interventions were described and classified in detail using a modified version of the TIDIeR checklist for complex interventions. Outcomes were described and classified using the Core Outcome Measures in Effectiveness Trials (COMET) taxonomy. Risk of bias was assessed using RoB2 and ROBINS-I.

**Results:**

The inclusion criteria were met by 103 articles. Four classes of acute care model were identified, acute-bed based care, hospital at home, emergency department in-reach and care home models. The field is dominated by small single centre randomised and non-randomised studies. Most studies were judged to be at risk of bias. A range of outcome measures were reported with little consistency between studies. Evidence of effectiveness was limited.

**Conclusion:**

Acute care models for older people living with frailty are heterogenous. The clinical effectiveness of these models cannot be conclusively established from the available evidence.

**Trial registration:**

PROSPERO registration (CRD42021279131).

**Supplementary Information:**

The online version contains supplementary material available at 10.1186/s12877-023-04373-4.

## Introduction

Population ageing and the increasing prevalence of long-term health conditions represent a significant challenge to many advanced health care systems [1]. Older people, particularly those living with frailty and multimorbidity, are at high risk of sudden health crisis necessitating urgent assessment to identify and treat causative conditions. The acute care pathway collectively defines the clinical processes employed to achieve this function. It typically comprises sequential assessment in community and hospital settings and culminates in emergency hospital admission when necessary.

Older people living with frailty are at high risk of adverse outcomes such as mortality [2] and have longer average lengths of hospital stay when accessing the acute care pathway [3]. The conversion rate from ED attendance to emergency admission is 3 times higher in people aged over 85 relative to people under 65 [4]. As older people represent a growing proportion of ED attendances the demand for hospital bed-based care is likely to rise [4]. This must be reconciled with downward trends in the number of acute hospital beds at the population level [5]. Improved integration between health and social care may help mitigate the impact of these changes to some degree but will not abrogate the need for hospital assessment and inpatient bed-based care in the context of sudden deterioration or severe illness [6]. Adaptations to the acute care pathway may improve the quality of care for older people while simultaneously reducing pressure on an increasingly congested acute care system.

These factors have collectively driven a rapid expansion of studies investigating models of care intended to mitigate the risk of hospital admission or avoid bed-based hospital care entirely [7]. Previous systematic reviews of acute care models for older people have focused on interventions located at specific points along the acute care pathway [8–10]. There has been a tendency to group interventions with different eligibility criteria and clinical processes. Differentiating models of care able to manage acute illness from those primarily engaged with rehabilitation and the functional consequence of resolving acute illness is not straightforward. This distinction is important as policy makers and commissioners look to maximise the efficiency of acute hospital bed use and find credible alternatives to acute inpatient care in the community.

It is possible that a more granular classification of the interventions may foster a greater understanding of which elements of the model drive effectiveness and highlight areas of best practice.

A systematic review was undertaken to describe and classify the range of acute care models designed to manage acute medical illness in older people with the objective of deriving a taxonomy of care models. The review also aimed to describe and classify the outcome measures used in studies investigating these models. A secondary objective was to determine whether the proposed taxonomy was useful in understanding any differences in observed outcomes between studies. We took the novel approach of including acute care models operating in hospital and community settings.

## Methods

### Study design

The systematic review was conducted using a two-step process. The first step was undertaken to describe and classify acute care models for older people and the outcome measures used to demonstrate their clinical effectiveness within the current literature. This information was used to create a taxonomy of care models accompanied by a narrative summary. No restrictions were placed on study design at this stage of the process.

The second step looked to describe the effectiveness of each model and restricted analysis to randomised controlled trials or observational studies with an experimental design (including non-randomised trials, cohort studies with comparator groups, before and after longitudinal studies). Previous systematic reviews and meta-analyses were not used to inform the taxonomy. Primary studies from relevant systematic reviews were included if they met the inclusion criteria. The systematic review was undertaken in accordance with the Preferred Reporting Items for Systematic Reviews and Meta-analyses (PRISMA) reporting guideline. The study protocol was registered with PROSPERO (CRD42021279131).

### Eligibility criteria and study selection

Inclusion and exclusion criteria were designed to incorporate interventions operating within the hospital and the community. An age threshold of 65 years was used to define care models for older people (mean age of study participants > 65 years. Mean age as opposed to a strict age threshold was employed to ensure care models accepting younger patients with frailty identified using alternative measures, such as validated frailty scores or multi-morbidity were not excluded.

The intervention needed to target acute medical illness or acute exacerbation of chronic disease. There is no consensus definition of acute care. To ensure a focus on acute care, study participants needed to be recruited within 48 h of presentation and the care model had to provide time limited episodes of care (up to 14 days). The requirement for time limited episodes of care was used as a criterion to exclude care models delivering ongoing chronic disease management after resolution of acute illness which were felt likely to employ different care processes and focus on different clinical outcomes. Recruitment direct from the ED was used as a proxy for recruitment within 48 h in studies where this metric was not reported. Community interventions were only included if they were able to provide a credible alternative to hospital bed-based care. This was defined as the capability to provide face-to-face review alongside access to hospital level treatments (eg intravenous treatments) and hospital level diagnostics (eg blood tests, imaging) at home.

A full list of inclusion and exclusion criteria is provided in Table 1.

**Table 1:** The Inclusion and exclusion criteria for the systematic review

	Inclusion	Exclusion
Study type	Taxonomy development I. Randomised and non-randomised controlled trials II. Prospective or retrospective cohort studies (with or without comparators) III. Study protocols	III. Systematic reviews IV. Narrative reviews
	Effectiveness I. Randomised and non-randomised controlled trials II. Observational studies with an experimental design	I. Systematic reviews II. Narrative reviews III. Observational studies without a comparator group IV. Study protocols without outcome data
Participants	I. I. Mean age of study participants over 65 years II. II. Acute medical illness or acute exacerbation of chronic disease	
Interventions	I. Targeted at individual person (not group interventions) II. Enrol patients within 48 h of presentation (in community or hospital setting) III. Provide time limited episodes of care (up to 14 days)	I. Community models that do not provide a replacement for acute bed-based care (no access to hospital level diagnostics or treatments) II. Outpatient ambulatory or home intravenous antibiotics services III. Reablement or transitional care services providing care after resolution or acute illness IV. Home hospice models V. Mental health, paediatric, surgical and obstetric models

### Data sources and searches

The search strategy comprised both MeSH terms and keyword text and was performed on 30^th^ September 2021 with no date restrictions. The search strategy is provided in Supplementary Table 1. The search was undertaken in 5 electronic databases (Ovid MEDLINE, Ovid Embase, Cumulative Index to Nursing and Allied Health Literature, Cochrane Database of systematic reviews, Cochrane Central Register of Controlled Trials). Hand reference list screening was carried out of all included articles. Systematic reviews were not included directly. All individual studies meeting the inclusion criteria contained within systematic reviews identified by the search were included.

Titles and abstracts were reviewed by two reviewers. (TK reviewed each and at-least one further review from CA, VK, CG, JR). Full-text records were obtained and reviewed against the eligibility criteria. Disagreements were resolved by a third reviewer (DL). Data extraction was undertaken by 1 reviewer (TK). A bespoke data extraction tool was adapted from the TIDIeR checklist to characterise each intervention [11]. Outcome measurements were classified using the Core Outcome Measures in Effectiveness Trials (COMET) taxonomy [12].

### Data extraction and quality assessment

Risk of bias was assessed using criteria from the Cochrane Handbook. Randomised controlled trials were assessed using RoB-2 tool [13] and observational studies were assessed using the ROBINS-I tool [14]. Risk of bias was assessed by 1 reviewer (TK).

### Data synthesis

Finding from included articles were grouped and summarised. Due to clinical heterogeneity between studies meta-analysis was not appropriate. A narrative synthesis of the results was undertaken. Visualisations were created using R statistical software (Version 1.3.1093, Vienna. Austria). The geographical location of included studies was mapped using the ggmap package. Source maps were obtained from *©* Stamen Design, under a Creative Commons Attribution (CC BY 3.0) license. Outcome areas and domains were plotted using the treemap package.

## Results

**Fig. 1:**
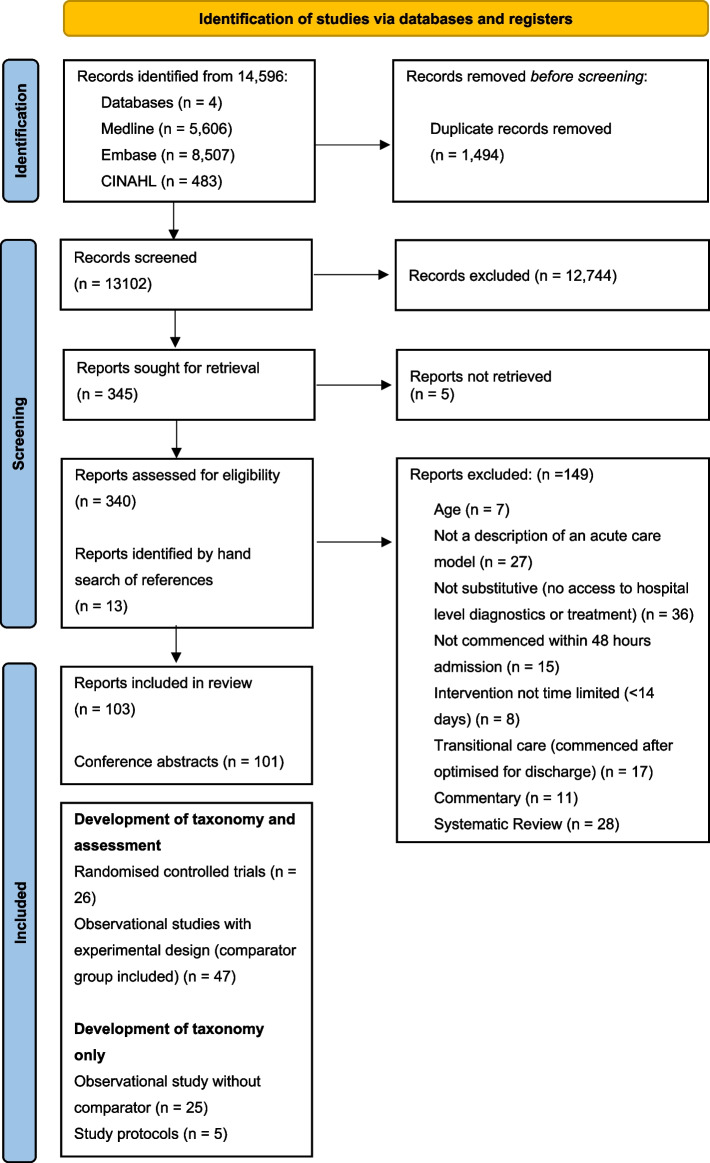
A PRISMA flow diagram for the studies screened and included in the systematic review. Legend: Studies were screened against the inclusion and exclusion criteria described in Table 1. Reasons for exclusion are provided

The initial search returned 13,102 relevant articles. Title and abstract screening identified 340 relevant articles for full text review. A total of 90 articles met the eligibility criteria. Hand searching of references identified 13 further articles. Therefore, 103 articles were included in the analysis (see Fig. 1). Identified articles were published between April 1991 and April 2021. This comprised 20 randomised controlled trials reported across 26 articles), 6 study protocols (results for 2 had been reported and were included), 38 observational studies with a comparator group reported across 51 articles, and 20 descriptive studies without a comparator group. The search identified 101 conference abstracts which did not contain sufficient information to adequately describe the model of care delivered. These abstracts were not used to inform the taxonomy.

### Taxonomy

**Fig. 2:**
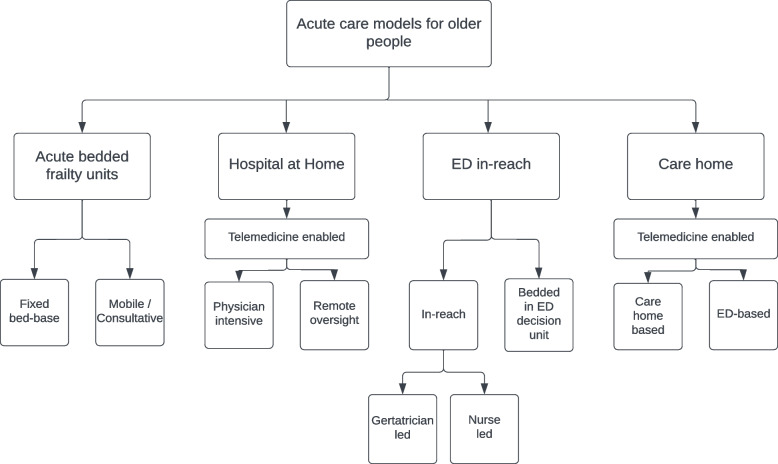
The Proposed taxonomy of acute care models for older people. Legend: The taxonomy was defined using key features of the care models; Care models were initially differentiated based on location. Acute bedded frailty units operated from a fixed bed base or offering consultation to general medical wards. Hospital at home models were differentiated based on their use of telemedicine. Physician intensive models used face to face review at home as standard. Remote oversight models were primarily delivered by specialist nurses with care supported provided remotely by physicians on a selective basis. Emergency Department in reach models could be differentiated by their staffing model. Nurse led care coordination without direct input from a dedicated geriatrician or care delivered by geriatricians within the Emergency Department. Care home models were differentiated by their primary location of activity, either services offered within the care home or adaptations to the care pathway following transfer to the Emergency Department

**Fig. 3:**
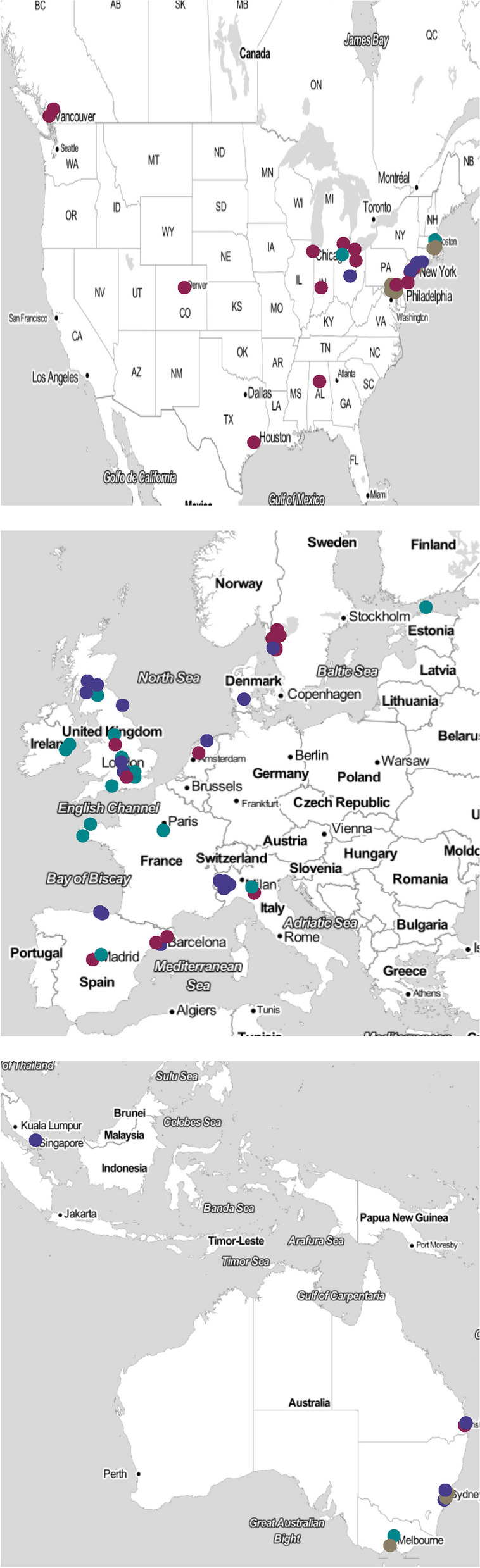
A map identifying the countries where the included studies were based. Legend. The map shows the location of included studies identifying: Colours to denote the care model type as defined by the taxonomy. Brown dots represent Hospital at Home models, Violet dots represents bedded Acute Frailty Units. Purple dots Emergency Department in-reach models. Green dots care models. Source maps were obtained from © Stamen Design, under a Creative Commons Attribution (CC BY 3.0) license

The articles could be broadly categorised into four groups based on the model of care they described. These included: bedded acute frailty units (AFU), Hospital at Home models (HaH), ED based in-reach models and acute care home models, see Fig. 2. A detailed description of the interventions described in each individual study is provided in Supplementary Table 2. The geographical location of included studies is provided in Fig. 3.

### Bedded acute frailty units models

The provision of tailored bed-based in-patient care for frail adults as a direct alternative to treatment on a general medical ward was described in 32 articles derived from 24 studies. This included 8 articles [15–22] reporting results from 6 randomised controlled trials, 1 trial protocol without results [23], 11 observational studies with a comparator group reported across 15 articles [24–38] and 8 descriptive studies without a comparator [39–46]. A detailed description of the care models is provided in Supplementary Table 2A.

The AFU care model has a strong focus on maintaining and restoring function, but in contrast to a rehabilitation ward intervenes prior to full resolution of acute illness. A range of names were used to identify care models with similar underlying approaches, including Acute Frailty units (AFU), Acute Care for Elders (ACE) units and CGA units. Generic descriptions of the model frequently reference four core components, patient centred care, specifically designed environments, review of medical care and early discharge planning as key characteristics of the model. There was considerable variation in how these shared high-level objectives were operationalised within individual care models.

Treatment was delivered within a geographically distinct bedded unit in 20 studies [15–19, 21–24, 26–32, 34, 35, 38, 39, 42, 44–46], of which 7 specifically reported adaptations to optimise the environment for older people [15, 17, 18, 23, 24, 39, 41]. The mean number of beds in each unit was 18 (SD 8). The number of beds was not reported in 3 studies [25, 41, 46]. A mobile model providing specialist consultations to patients within general medical bed was described in 3 studies [20, 33, 36] (and an integrated service with variable bed capacity operating within an acute medical unit in 1 study [45].

Eligibility criteria were heterogenous. Age criteria were reported in studies describing 20 care models [15–22, 25–33, 35–39, 41, 42, 44, 45]. Descriptions of the process of patient referral and how eligibility criteria were implemented in practice were uncommon. The presence of additional criteria such as functional impairment or specific geriatric conditions were frequently reported, but it was not possible to establish how these criteria were operationalised. The use of validated frailty assessment tools to define eligible patients were reported in 1 study (reported across 5 articles) [26, 28–31]. Patients from residential care homes were excluded in 2 studies [18, 21]. Bed availability was cited as a common determinant of receiving treatment on the AFU.

### Hospital at home models

Hospital at home (HaH) models describe the provision of acute medical care within a person’s usual place of residence. The care model aims to replicate acute bed-based care and operate under the assumption that care would be delivered in an acute hospital setting if the model were absent. HaH models were described in 37 articles derived from 27 studies. This included 16 articles [47–62] reporting results from 12 randomised controlled, 2 protocols (of which 1 had reported results and was included) [63, 64], 9 observational studies with a comparator group reported across 15 articles [65–78] and 4 descriptive studies without a comparator group [79–82]. A detailed description of the care models is provided in Supplementary Table 2B.

There was significant clinical heterogeneity between included HaH models. The model accommodated patients with unselected acute medical illness in 31 studies and specific disease groups in 7 studies (decompensated heart failure = 3 [57, 58, 62], COPD = 4 [47, 51, 52, 70, 79]).

Eligibility criteria to define suitability for HaH care were heterogenous. All included studies made the intention to act as an alternative to hospital bed-based care explicit. Clinical discretion exercised by the HaH team was the arbiter of the appropriateness and safety of HaH care in all the identified studies. No standardised approach to assessment was identified and it was not possible to reliably determine the acuity of included patients from the reported data. The majority of HaH studies specifically targeted adults over the age of 65. In models open to adults of all ages, the mean age of participants was over 65 in all cases. Care home residents were excluded in 9 studies [53, 58, 59, 63, 67, 73–75, 80].

Care was led by a geriatrician in 6 studies, [47, 59, 61, 62, 73, 78] by a general internal medicine physician in 29 studies and a primary care physician in 2 studies [60, 83]. The intensity of physician and nursing involvement varied substantially. Physician involvement ranged from multiple daily physical home visits to remote oversight without direct physical assessment. Specific out-of-hours arrangements were reported in 12 studies reported across 19 articles [47, 53–55, 61, 62, 65, 67–69, 71, 72, 74–77, 81–83]. The use of telemedicine was described in 5 studies reported across 11 articles [47, 65, 66, 68, 69, 71, 72, 74–77]. Reporting of the study intervention was often restricted to a description of standardised operating procedure. The frequency of assessment achieved in practice was reported in 6 studies [52, 53, 58, 74, 75, 81] and the proportion of patients receiving specific treatments was reported in 3 studies [47, 53, 80].

### ED in-reach models

ED in-reach models aim to optimise processes of care for older people in the ED. The care models typically provide care coordination and elements of CGA to reduce the likelihood of admission to acute-bed based care. ED in-reach models were described in 28 studies describing 27 care models. This included 2 randomised controlled trials, [84, 85] 1 randomised controlled trial protocol without results [86], 12 observational studies with a comparator group [87–98] and 13 descriptive studies without a comparator group [99–111]. A detailed description of the care models is provided in Supplementary Table 2C.

Two distinct approaches to the operational design of services were evident. One approach, described in 11 studies, involved the use of bedded areas located within ED clinical decision units (alternatively referred to as ED short stay units) to provide elements of CGA to older patients who required additional assessment and investigation before a decision regarding acute medical admission could be reached [87, 89–91, 94, 96, 104, 105, 107, 109, 111].

An alternative approach, described in 20 studies, involved the provision of elements of CGA directly within the ED. CGA was undertaken by a geriatrician in 10 care models [84, 88, 97, 100–103, 108, 110] and by specially trained nurses in 7 care models [85, 86, 92, 93, 95, 98, 99, 106]. Studies of this care model frequently cited a reduction in the number of avoidable medical admissions as the primary motivation for the service. The distinction between avoidable and unavoidable admissions was poorly defined.

Eligibility criteria were heterogenous. Age criteria were reported in 13 care models [84, 88, 91, 93–95, 98, 99, 102–104, 106, 108, 112]. The use of validated frailty assessment tools to define eligible patients were reported in 5 care models [84, 86, 92, 99, 106]. Care home residents were excluded in 3 studies [86, 91, 94]. Eligibility criteria were not reported in 5 studies [87, 89, 91, 109, 110]. A variety of approaches were adopted to identifying potentially eligible patients in the ED. Screening of all patients attending the ED was reported in 3 studies [84, 88, 93]. The service was accessed by a referral from the ED team in 11 care models [89, 90, 92, 95, 98–101, 104, 109, 110]. The process of referral and patient selection were not consistently reported.

### Acute care home models

Models targeting care home residents were reported in 5 studies. All 5 studies had an observational design [113–117]. Two categories of intervention were described. The first involved the presence of dedicated staff trained in acute care present with the care home [113, 117]. These staff had the ability to deliver acute interventions in the care home. Privileged access was given to the on-call ED physician in both models (augmented by telemedicine in one study) [117]. The process which triggered assessment by the on-site team were not defined. A detailed description of the care models is provided in Supplementary Table 2D.

An alternative model involved a hospital-based team providing out-reach to care homes and early assessment of care home resident presenting to ED. Both care models in this category also had the capability to provide ongoing acute care in the care home when required. This was achieved by a geriatrician-led team with the option to provide daily visits in one model [116] and a specialist ED nursing team in the other [114, 115].

### Outcome measurements

Outcomes were classified using the COMET taxonomy. Outcomes were reported across 6 core areas and 15 domains. Mortality was reported (in isolation or as part of a composite outcome) in 35 studies, the reporting time horizon ranged from in-hospital mortality to 1 year. Life impact was reported 27 studies, this included measurement of physical function in 21 studies and cognitive function in 6 studies. The tools used to measure physical function and the time horizons of assessment varied.

Resource use was the most reported core outcome measure. Studies frequently described multiple outcome domains related to resource use. The average length of stay was reported in 34 studies and re-admission rate in 39 studies. Readmission rates were reported over a range of time horizons 30 days to 1 year. Care home admission were reported (in isolation of as part of a composite outcome) in 14 studies over a time horizon of 30 days to 6 months. Economic analysis was reported in 19 studies. Adverse events were reported in 22 studies. A detailed summary of the outcome domains, methods of measurement and associated time horizons is provided in Supplementary Table 3.

**Fig. 4:**
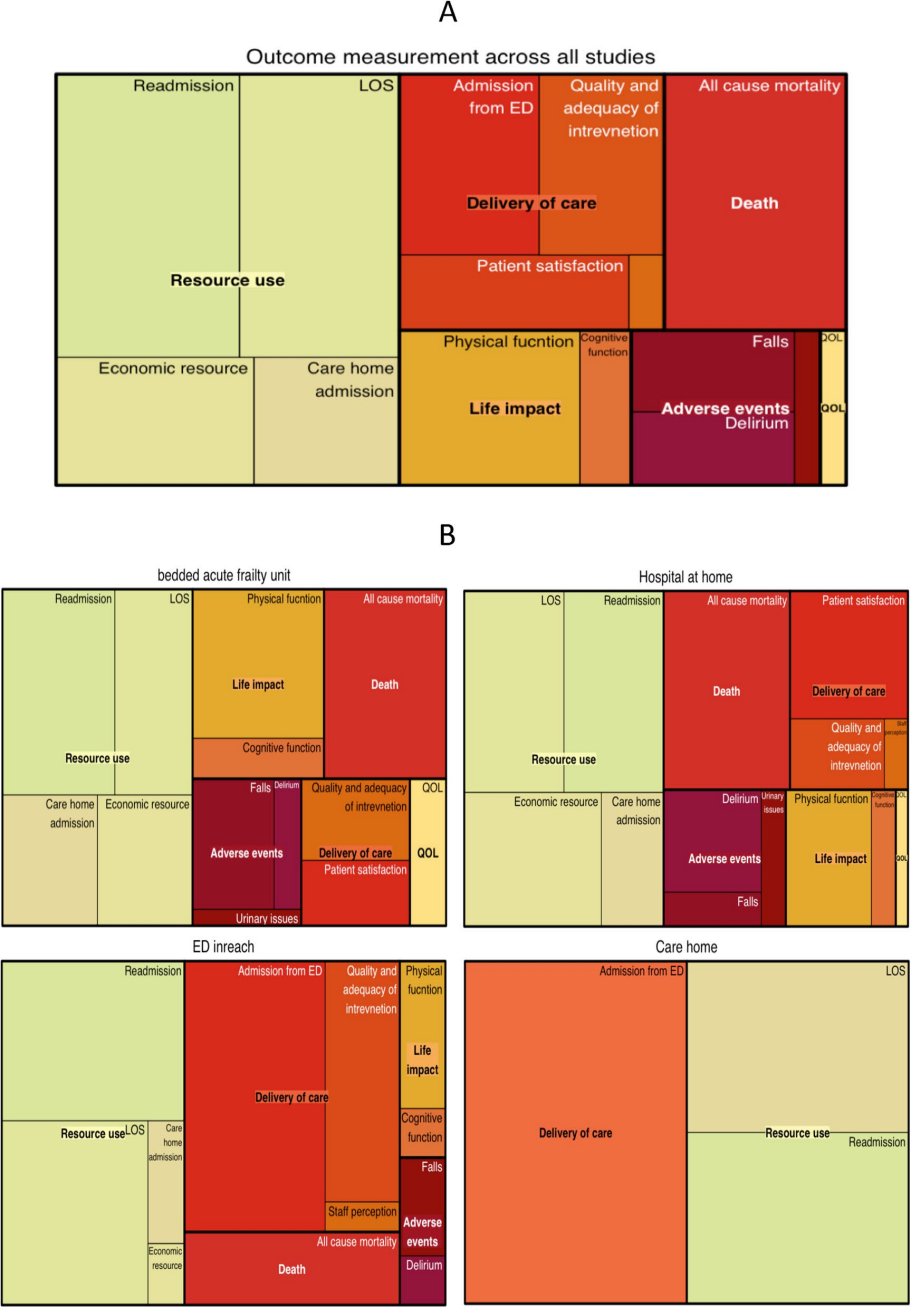
Tree diagrams: **A** tree diagrams representing the relative proportion of outcomes reported in all studies. **B** Tree diagrams representing the relative proportion of studies by study group. Legend. * Treemap representing hierarchical outcome data using nested rectangles. Large rectangle represent core outcome areas, smaller rectangular tiles within each core outcome area represent outcome domains. Each rectangle has an area proportional to the frequency reported within included studies. All studies *n* = 103, Bedded acute frailty unit *n* = 32, Hospital at Home *n* = 38, ED in reach models *n* = 28, Care home *n* = 5

The relative frequency with which the outcome domains were reported across all studies is provided in Fig. 4A and stratified by care model in Fig. 4B. Outcomes reported by bedded AFU and HaH were broadly similar, although AFU more commonly reported outcomes related to physical function. Economic analysis was less prevalent in studies investigating ED in-reach models. A focus on aspects of care delivery, such as disposition from the ED and analysis of clinical processes relevant to the quality and adequacy of intervention were more common in studies evaluating ED in-reach.

### Effectiveness

**Fig. 5:**
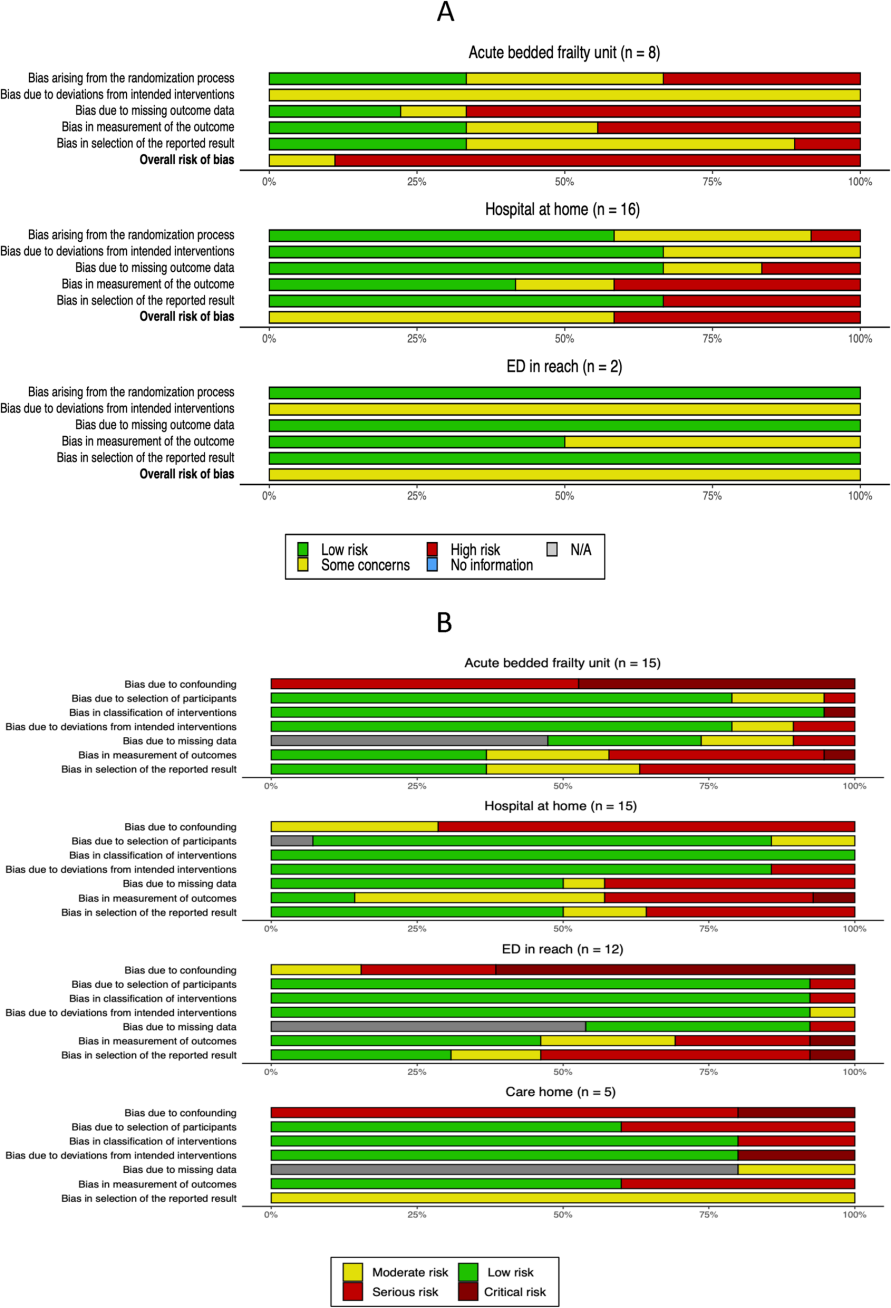
Summary of bias assessments. **A** Summary of randomised controlled studies using RoB2 tool. **B** Summary of non-randomised studies using ROBINS-I tool

Clinical heterogeneity amongst the care models identified and disparity in the outcomes measured used to evaluate the care models precluded meta-analysis. Risk of bias was assessed for each study. Aggregated results of the domain-based risk of bias assessment tools are provided in Fig. 5 and the results of individual study assessments are provided in Supplementary Table 4.

The nature of the intervention precluded blinding of participants or personnel to group allocation in all included randomised controlled trials. Partial blinding of outcome assessment was reported in one study investigating the effectiveness of bedded AFUs [17] and assessment was unblinded in the remainder. Blinding during outcome assessment was reported in 4 randomised controlled trials investigating HaH [47, 52, 59, 60]. Outcome assessment was unblinded in both randomised controlled trials investigating ED in-reach models [84, 85]. All the studies investigating bedded AFUs were undertaken in single sites which may have led to contamination of the control arm. This would be anticipated to favour the null hypothesis [15, 16, 18–22]. Contamination of the control arm was less likely in HaH models delivered by distinct clinical teams.

All included observational studies were at serious or critical risk of confounding. The decision to manage patients in the intervention arm is likely to have been selective, based on clinical judgment informed by pre-intervention clinical characteristics. Only 5 studies employed robust statistical techniques to control for confounding [65, 67, 69, 78, 92]. Residual confounding from unmeasured prognostic factors posed at risk of bias all included observational studies.

### Effectiveness of acute care models

#### Bedded acute frailty unit models

No statistical difference in primary outcome was observed in 2 randomised controlled trials (reported across 3 articles) of specialist bed-based care for unselected older medical patients, 1 study measured the composite outcome of death, severe dependence and psychological well-being [15] and the other physical function at 3 months following discharge [19]. A planned cost-analysis demonstrated no difference in the total cost of admission between groups [16]. A single centre randomised controlled trial comparing a specialist unit for acutely unwell patients with cognitive impairment with usual care demonstrated no statistical difference in the composite outcome of days at home [17]. All included observational studies were judged to be at critical or serious risk of bias.

#### Hospital at home models

The largest randomised controlled trial included 1055 participants [59]. The study was designed to recruit to the HaH intervention at a ratio of 2:1. A significant number of participants moved from the control to the intervention arm due to operational pressures within the hospital. The study found no difference in the primary outcome of living at home at 6 months (the inverse of death or long-term residential care) [59]. The remaining 11 trials (reported across 15 articles) had smaller sample sizes (mean 81 participants, SD 33). One randomised controlled trial (2 articles) reported a statistically significant reduction in the rate of adverse events [50] and favourable functional outcomes in the group allocated to HaH care [49].

HaH care for older people with decompensated heart failure was investigated in 2 randomised controlled trials, 1 reported no difference in mortality or readmission at 6 months [62] and 1 no difference in mortality or readmission at 12 months [57]. HaH care for older people with an acute exacerbation of COPD was investigated in 2 randomised controlled trials, 1 reported a statistically significant reduction in readmissions at 6 months and no difference in mortality at 6 months [47] and 1 reported lower costs at 90 days, driven by shorted length of stay in the HaH group, with no difference in mortality or readmission rate at 90 days [52]. Economic analysis determined HaH was associated with lower costs in 1 randomised controlled trial of participants with unselected medical-illness [53]. Nested analysis of patient and carer satisfaction was included in 5 trials [47, 52, 53, 59, 62] in 3 trials the findings were reported in separate articles [51, 55, 66]. All showed an increase in measures of patient satisfaction in the HaH intervention group.

One randomised trial compared two contrasting models of HaH. The study arms compared HaH care led by primary care physicians with care led by hospital specialists [60]. Those in the hospital specialist arm were initially assessed in the ED and discharged within 4 h of assessment with a home-based care plan. The hospital specialist team did not undertake home visits. Those in the primary care physician arm received care exclusively at home. In both arms the plan care was delivered by a dedicated HaH nursing team. The primary care physician model was a associated with a statistically significant reduction in hospital admission at 7 days. A series of articles published as part of a non-randomised controlled trial [75] reported a reduction in length of admission, [75] reduced levels of carer stress [71] and no difference in physical function [72] in the HaH group.

#### ED in reach models

No statistical difference in the primary outcome measure was observed in 2 randomised controlled trials investigating ED in-reach models. In one study the provision of geriatrician lead CGA to patients aged over 75 with a clinical frailty scale (CFS) of 4 or above did not affect cumulative length of stay over a 1 year follow up period [84]. A randomised controlled trial investigating provision of nurse-led care coordination in the ED found no significant effect on the rate of hospital admission [85]. Uncontrolled before and after studies were a common methodological approach to the assessment of ED in-reach models, employed in 5 studies. All included observational studies were judged to be at serious risk of bias.

## Discussion

This systematic review provides a summary and classification of acute care models for older people living with frailty and an assessment of effectiveness based on current published evidence. The care models identified could be broadly differentiated by the location within the acute care pathway at which they operate. This generic classification provides a degree of structure to a large and complicated field of research, sensitive to the fact that relevant interventions have emerged across hospital and community settings. The spectrum of outcomes reported and differing approaches to measurement suggest consensus on how best to determine the effectiveness of these care models has yet to emerge.

The clinical effectiveness of acute care models for older people was difficult to determine from the available studies. The number of participants within each trial was small. The risk of confounding by indication was pervasive amongst observational studies and statistical techniques to control for cofounding were generally absent or inadequate. These methodological limitations prevented meaningful comparisons of the impact on outcomes between care models. There is a paucity of contemporary data on the effectiveness of acute care models for older people. Some of the most influential studies were conducted over two decades ago. This raises the concern that the clinical processes employed may now be obsolete.

Complex interventions, such as acute care models for older people are often difficult to characterise. The detailed summary of individual interventions provided within this review highlights the contrasting approaches adopted by services under the same umbrella.

Few studies adopted a structured approach to defining the intervention under investigation and the descriptions provided varied in depth and quality. The nature of care provided in the usual care arm of comparative studies was equally difficult to define. The absence of consistent inclusion and exclusion criteria or knowledge of how criteria were operationalised makes it difficult to discern the population targeted by each intervention. Assignment often incorporated a subjective assessment by an individual clinician acting as gatekeeper. Thresholds for admission and discharge are not standardised and risk tolerance may vary at the individual, hospital and system level. This is particularly pertinent to studies investigating the role of HaH and ED in-reach models, predicated on the assumption that care would inevitably require in-patient bed-based care if the intervention was absent. This assumption is inherently difficult to substantiate. All the HaH models included in this systematic review had access to hospital level diagnostics and interventions but the proportion of patients receiving these interventions were inconsistently reported. This obfuscates an objective assessment of acuity and whether hospital admission was warranted.

### Comparison with previous literature

Clinical heterogeneity in the studies included in previous systematic reviews and the absence of universally accepted definitions for the care models investigated cloud interpretation of the existing literature. The diverse range of approaches to patient selection, operational design and outcome measurement highlighted in this review suggests caution is warranted when pooling studies in this subject area.

Several systematic reviews investigating acute care models for older people have focused the delivery of comprehensive geriatric assessment (CGA) [8]. CGA involves multidimensional assessment with particular attention on the functional consequences of illness [118]. CGA has been shown to increase the likelihood of being alive or returning to home at 3 to 12 months follow up amongst older patients admitted to hospital with acute illness [8]. Meta-analysis of CGA delivered in bed-based frailty units found a lower risk of functional decline, a higher likelihood of living at home after discharge and no differences in mortality [119]. CGA delivered in bed-based frailty units may also reduce the incidence of adverse events such as falls, delirium and pressure sores at discharge [10]. The inclusion of interventions delivered on rehabilitation wards, and patients with surgical and orthopaedic presentations in previous systematic reviews limits generalisation to care models employed at earlier time points in the acute care pathway. The available literature suggests alternatives to usual bed-based care incorporating CGA may be of benefit but offers little to guide how these services should be designed and implemented. When inclusion is limited to interventions employed within 48 h of presentation the evidence of effectiveness is less compelling. This is important given the benefit of CGA is cited as the primary motivation for operational models located upstream in the acute care pathway [120].

HaH models have also been the subject of systematic review and meta-analysis. A Cochrane review of admission avoidance HaH identified ten randomised controlled trials including 1333 participants of which 850 were included in individual patient level meta-analysis [121]. The analysis demonstrated a significant reduction in mortality at 6 months (adjusted HR 0.62, 95% CI 0.45–0.87). A more recent systematic review and meta-analysis found patients managed in HaH following discharge from the ED had a lower risk of admission to institutional care (RR 0.16 95% CI 0.03–0.74) and no difference in mortality (RR 0.84 95% CI 0.6–1.2) [122]. These systematic reviews pooled results from studies investigating HaH in the context of a diverse range of conditions including stroke, cellulitis, fractures and respiratory illness which would be expected to employ very different clinical processes. Applying a more restrictive approach to study inclusion, by only including HaH models with access to hospital level diagnostics and treatments allows greater confidence in the assertion that the HaH models included in the current review offered a true alternative to hospital admission.

### Implications for policy and future research

The provision of acute care models for older people are predicated on a logic model rather than empirical evidence of benefit. Further large and rigorously constructed randomised controlled trials may strengthen the evidence base but may not be the most effectual method of influencing local decisions on service provision or the direction of policy.

Research in acute care delivery is complicated by a need to maintain operational performance. Amongst the studies identified, bed availability and restricted operational hours frequently resulted in a large differential between the number of potentially eligible participants and the number of patients ultimately included. Practical considerations aside, the outcomes of interventional studies are likely to be highly dependent on local context and external factors which influence generalisability.

Knowledge in this subject area may be enhanced by developing a consistent approach to outcome reporting and measurement, ideally incorporating the priorities and preferences of patients. Mortality may not be the most appropriate metric of effectiveness given a significant proportion of older people living with frailty requiring acute care for medical illness are entering the last 12 months of life [123]. Current models of acute care infrequently establish and record individual preferences in relation to location of care in the event of acute medical illness or preferred location of death amongst older people [124]. A narrow focus on clinical and operational outcomes may simplify study design, facilitate comparisons and provide reassurance around safety but risks ignoring other aspects of care, such as quality of life, which may be more meaningful from the patient perspective.

Given the complexity of the intervention, an understanding of the processes and behaviours which drive successful models may be best approached from a qualitative research paradigm.

### Strength and limitations

The primary objective of this systematic review was to describe and categorise acute care models for older people and highlight variation in the outcome measures used to assess them. An extensive search strategy inclusive of the grey literature and indifferent to methodological design was purposefully employed in order to capture a comprehensive representation of the range of models in operation. Every acute hospital encounters older people living with frailty and the potential for variation in approach is vast. Only a small fraction of care models delivered in practice are reported in the literature. The practice of publishing multiple articles from the same original study was relatively common, particularly in literature pertaining to acute bed-based care and HaH models. The account provided is therefore susceptible to both publication and outcome reporting bias.

## Conclusion

Acute care models for older people living with frailty are heterogenous. The clinical effectiveness of these models cannot be conclusively established from the available evidence.

### Supplementary Information

**Additional file 1. **

**Additional file 2. **

**Additional file 3. **

**Additional file 4. **

## Data Availability

The datasets used and/or analysed during the current study available from the corresponding author on reasonable request.
