# Supplementary material for: Acute care models for older people living with frailty: a systematic review and taxonomy
Source: BMC Geriatr. 2023 Dec 5;23:809. doi: 10.1186/s12877-023-04373-4 (PMC10699071; doi:10.1186/s12877-023-04373-4)
Supplement: Supplementary file 1 — Additional file 1. [file 12877_2023_4373_MOESM1_ESM.docx]

Supplementary material 1

Search strategy

Ovid MEDLINE(R)

1 Geriatric Assessment/

2 health services/ or health services for the aged/

3 Home Care Services, Hospital-Based/

4 Hospital Units/

5 Outcome Assessment, Health Care/

6 "Delivery of Health Care"/

7 (hospital* adj2 at home) or home hospitali#ation or home-based or "at home" or homecare or home treatment or admission) or (admission adj2 avoid*).mp. [mp=title, abstract, original title, name of substance word, subject heading word, floating sub-heading word, keyword heading word, organism supplementary concept word, protocol supplementary concept word, rare disease supplementary concept word, unique identifier, synonyms]

8 ((hospital-level or hospital level or substitutive) adj2 care*).mp. [mp=title, abstract, original title, name of substance word, subject heading word, floating sub-heading word, keyword heading word, organism supplementary concept word, protocol supplementary concept word, rare disease supplementary concept word, unique identifier, synonyms]

9 comprehensive geriatric assessment.mp.

10 Frailty/ or Frail Elderly/ or Aged/ or "Aged, 80 and over"/

11 3 or 4 or 5 or 6 or 7 or 8 or 9

12 10 and 11

13 1 or 2 or 12

14 Acute Disease

15 Emergency Service, Hospital

16 Emergencies

17 Patient Admission

18 Hospitalization

19 14 or 15 or 16 or 17 or 18

20 13 and 19

Legend: Ovid Medline search terms
