# Supplementary material for: Acute care models for older people living with frailty: a systematic review and taxonomy
Source: BMC Geriatr. 2023 Dec 5;23:809. doi: 10.1186/s12877-023-04373-4 (PMC10699071; doi:10.1186/s12877-023-04373-4)
Supplement: Supplementary file 2 — Additional file 2. [file 12877_2023_4373_MOESM2_ESM.docx]

Supplementary table 2

The tables provide a description of the care models of included studies. Bedded Acute Frailty units, Hospital at Home, ED in-reach and Care home models are reported in separate tables. Each row contains a description of the care model. Studies reporting the same care model are reported on the same row.

2A; Bedded Acute Frailty Unit care models

| **Author** | **Study type** | **Where:** | **Environmental  adaptions** | **Source of referral** | **Process of referral** | **Eligibility Age** | **Eligibility Clinical** | **Population** | **Who: Team and training** | **Standard of care** | **Frequency of assessment and intervention (as achieved)** | **Proportion of eligible patients assessed** |
| --- | --- | --- | --- | --- | --- | --- | --- | --- | --- | --- | --- | --- |
| Abdalla et al [24] | Observational cohort | 9 beds. | Yes  non-skid flooring, low beds, low noise policy | ED | Not reported | None | Stated selected for frailty based on "ED screening for risk of developing geriatric syndromes" and "Medically stable". | Mean age; 78 Clinical  characteristics not reported | Specially trained nursing staff. Nature of training included teaching on delirium detection, skin integrity, sleep patterns. | Early access to physical therapy and mobilisation. Provision of appropriate equipment, use of bedside commodes, daily medication review and avoiding use of urethral catheters. | Not reported | Not reported |
| Abisheganaden et al [25] | Observational cohot | Not reported | Not reported | Not reported | Not reported | >65 | Study focused on sub-group of patients with pneumonia classified retrospectively using DSM | Mean age; 84  38% of patients had dementia | Geriatrician led. Specialist nurses . Access to physio, OT, SLT, SW. | CGA by interdisciplinary teams led by geriatrician. | Frequency not reported, described "Regular" team meetings to facilitate discharge planning. Routine assessment of swallowing and appropriate feeding modifications | Not reported |
| Ahmed et al [39] | Before and after study (uncontrolled) | 14 beds. | Yes  Environment adaptions to reduce falls risk.  Nature of adaptations Not described | ED | Direct admission from geriatric medicine OP and home visit  Referral process not reported. Described as "a random event", depending on bed availability and ED/referring physician preference; not all persons older than age 70 are sent to the unit. | > 70 or  > 65 co-morbid | Multiple comorbidity not specifically defined. No other selection criteria applied related to frailty. | Mean age; not reported  Reported patient mix by speciality No other clinical characteristics | Geriatrician led. An internal medicine resident, geriatric fellow, and/or geriatric nurse practitioner assist with medical care delivery. Access to physiotherapy, OT, SW, SLT. Assistance from volunteers. | Standardized admission order set, applicable to all disease processes related to physiotherapy and diet. Medical interventions restricted between 11 pm and 5 am, unless there is an emergency. | Frequency of medical assessment and multi-disciplinary rounds not reported. AF | Not reported |
| Asplund et al [15] | Randomised controlled trial | 11 beds. | Yes  Environmental adaptions to reduce falls risk. Not specifically described | ED | Random allocation for purposes of study. | > 70 | No other selection criteria applied related to frailty. | Mean age; 81  Significant proportion cardiac issues (25% myocardial infarction, 25% angina) | Geriatrician led. Access to physiotherapy and OT. No access to SW. | Consultants from both the geriatric and medical departments had joint responsibility for medical care | Not reported | Randomization was temporarily sus- pended if no capacity. Proportion of potentially eligible and duration of active recruitment not reported |
| Chittok et al [27] | Observational cohort | 21 beds | Not reported | ED | Not reported. Bed availability key determinant. No description of how bed allocation was prioritised when at capacity. | > 75 | No other selection criteria applied related to frailty. | Mean age; 83  Dementia and cognitive impairment in 10%  Independently mobile in 10% | Internal medicine, a hospitalist family practice, or a community family practice service provided medical care. Criteria for admission under each medical team not described. The same physicians provided care in ACE and usual care. | Early nursing, physiotherapy, occupational therapy, and social work assessments are automatic in ACE but not mandatory in usual care | Two full multidisciplinary rounds plus three brief chart rounds every week. | 1,150 eligible admissions during study period. Treatment was provided on the AFU in 53% |
| Covinsky et al [16]  Landerfield et al [19] | Randomised controlled trial | 14 beds. | Not reported | Not reported | Not reported. Bed availability key determinant. No description of how bed allocation was prioritised when at capacity. | > 70 | No other selection criteria applied related to frailty. | Mean age; 80  62% able to perform 5 or more ADLs at the point of admission Chane in mental status or other neurological complaint in 10% | Physician led, specialty not defined. Access to physiotherapy, OT, SLT, SW and dietician. | Daily assessment by nurses of physical, cognitive, and psychosocial function using validated tools and protocols to improve self-care, continence, nutrition, mobility, sleep, skin care, mood, cognition | Not reported | 1143 eligible patients were not enrolled because beds were not available in both the intervention and usual-care units at the time of their admission |
| Ahlund et al [26]  Ekerstad et al [28, 29, 30, 31] | Non-randomised controlled trial | 2 units   48 beds total. | Not reported | ED | Telephone triage by geriatrician | > 75 | FRESH (frailty screening instrument). Five questions relating to tiredness, falls, endurance, needing support while shopping, and three or more visits to the emergency department in the past 12 months.  If two or more of these questions were answered with a yes, the patient was considered frail | Mean age; 86  mean FRESH frailty screening score 3.5 | Physician led. Specialists in internal medicine, family medicine, and/or geriatrics contributed. Specialized admission and discharge nurses. Access to physiotherapy, OT, SW. | Care characterized by a systematic, structured, interdisciplinary CGA and care with validated instruments and standardized evidence-based procedures. Specifics of validated tools not reported. | Daily team conference | Not reported |
| Flood et al [32] | Observational cohort | 25 beds | No | Not reported | Patients admitted under care of general medicine allocated bed on AFU ward or general medicine ward if above 70. Bed availability key determinant of location. No description of how bed allocation was prioritised when at capacity. | > 70 | No other selection criteria applied related to frailty | Mean age; 82  No clinical characteristics reported | Geriatrician led. Responsibility for patient remained with admitting team.   Access to physiotherapy, OT, SW, SLT and dietician Volunteers provided cognitive stimulation and mealtime assistance. | Nurse coordinator administered geriatric screens; coordinated care between all team members, including volunteers and outpatient providers; and ensured that the daily geriatric care plan was implemented.  Unit did not have formalized nurse-driven care protocols or order sets, but it did use evidence-based geriatric care processes known to reduce the risk of geriatric syndromes. | Geriatrician-led rounds (Monday-Friday) | Only 39% of those on the ACE unit were aged >70. |
| Goldberg et al [17] | Randomised controlled trial | 28 beds | Yes   Adapted environment for patients with cognitive impairment. | AMU | Referred by physician on AMU following admission for acute medical illness. | > 65 | "confusion" identified bp physician on AMU (Used the term “confusion” as due to overlap between delirium and dementia acute population) | Median age 85;  Detailed description of degree of cognitive impairment. | Three nurses, an occupational therapist with expertise in mental health. Physiotherapy, SLTM OT. Healthcare assistants worked as activities coordinators | Staff were trained in recognition and management of delirium and dementia and the delivery of person cantered dementia care.  Consultant geriatricians on the ward had a special interest in delirium and dementia and wrote thorough discharge letters to family  Delirium prevention measures included careful diagnostic and drug review and early mobilisation | Not reported specifically. "access to standard medical and mental health services, rehabilitation, and intermediate and social care"   Twice weekly visits from a psychiatrist.  Frequency of specific interventions and processes descried in detail eg, 52% formal cognitive assessment, 81% collateral functional history | 1455 patients put through the allocation algorithm, 844 randomised. 71% of those allocated to the intervention arm received intervention. |
| Harris et al [18] | Randomised controlled trial | 14 beds. | Yes  Space for assessment of the activities of daily living and mobility problems. | ED | Decision for medical admission made by ED team. Study cases were identified each morning by reference to triage lists and the relevant medical notes using the eligbility criteria | > 70 | No other selection criteria applied related to frailty.   Excluded  Care home residents | Mean age; 79  50% of patients issues with cardiovascular system | Not directly stated whether geriatrician led.  Higher level of nursing staff physiotherapist and OT but exact staffing details not reported. | Specific care processes not reported | Not reported | Not reported |
| Hung et al [33] | Observational cohort | Mobile unit. | NA | NA | Patient known to OP geriatric medicine team were automatically reviewed following admission to hospital. The process of referral for patient not in this group is not reported | >70 | Under the care of geriatrician ambulatory clinic prior to admission. No other selection criteria applied related to frailty | Mean age; 85  Most common diagnosis pneumonia, falls and syncope.  Dementia 45.1% Delirium on presentation 22.5% | Geriatrician led. Geriatric fellow. Nurses with specialist training. Access to physiotherapy, OT, SLT, SW. | Specific care processes not reported | Team met daily in the morning (and afternoon if necessary) to discuss each patient’s care. Patients were assessed within 24-48 hours after admission | Analysis of a specific sub-group drawn from population known to OP geriatric clinic. . Reported capacity to assess approximately 500 admissions a year. How many assessments conducted not reported |
| Meschi et al [42] | Observational cohort (no comparator) | 14 beds | No | ED | Activated in response to high demand. Selection based on on "cumulative deficit" model through a comprehensive history and examination. How patients were targeted for comprehensive evaluation in not reported. | > 65 | "Targeted older patients with multimorbidity and high health care utilisation". Predicted LOS > 36 hours < 4 days. No specific written criteria provided. | Not reported | General internal medicine led supported by residents. Nurse patient ratio 1:6. | Not reported | Not reported | Not reported |
| Naughton et al [20] | Randomised controlled trial | Mobile  Geriatrician delivered care general medical ward | No | ED | Randomised in the ED after decision to admit had been made. Intervention arm admitted under the care of a geriatrician. Control arm admitted under general internist on call. | > 70 | All patients admitted to medicine from ED who did not receive regular care from an attending internist.   Excluded   Admitted to ICU Surgical | Mean age; 81  Patient level diagnoses not reported. Stated most common diagnosis CHF | Geriatrician led. Supported by a SW. Nurse clinical specialist and physiotherapist involved as required. | Admitted under care of a geriatrician. Attending responsibility rotated on a monthly basis. " team systematically, consistently, and routinely evaluated the patients’ mental status, psychosocial condition, functional status, and Medical condition to determine" | Team conferences two or three times per week. | Number of eligible patients not reported. 141 were randomised of which 25 were later deemed ineligible. |
| Jayadevappa et al [34] | Observational cohort | 36 beds | Not reported | ED | All older patients without a private physician admitted to unit direct from ED. Alternatively, attending physician could choose to refer to AFU. | >65 | "Non-critical " patients. | Mean age 80;  Diagnoses not reported | Geriatrician led. Specialist geriatric nurses. Pharmacist. Social worker. Similar nurse to patient ratio as general medical ward (5:1) | Not reported | Not reported | Not reported |
| Lin et al [41] | Observational cohot | Not reported. | Yes   Adaptions to optimse for older patients. Not specifically described. | ED | Determined at the discretion of the ED clinician. When the bed is available, patients would be admitted directly to the unit. If unavailable to admitted to a transitional care unit if GEMU bed is unavailable. Details of selection criteria not provided. | >75 | Evidence of "functional impairment with multi-morbidity". | Mean age; 91 | Access to physiotherapy, OT, SW, SLT and dietician Volunteers provided cognitive stimulation and mealtime assistance. | CGA completed by trained case managers. The case managers would provide the CGA information for team members to make a care plan separately after the evaluation. Case managers performed various functional assessments for all patients using validated tools | CGA in the first 72 hours of admissions follow-up CGA was performed before hospital discharge. | Not reported |
| Salinas et al [35] | Observational cohot | 10 beds | Not reported | ED. | Patients admitted to AFU or the general ward by ED without medical triage by AFU team.   Not reported. Bed availability key determinant. No description of how bed allocation was prioritised when at capacity. | > 65 | No other selection criteria applied related to frailty.   Exclusion  Dependent for all basic activities of daily living before admission | Mean age; 80  Categories for primary diagnosis reported.  26.5% had respiratory pathology | Geriatrician led. Nurses with geriatric specialist training. | Functional evaluation, early rehabilitation, promotion of self-care, neurosensory stimulation, orientation for the family and/or caregiver. Nursing interventions included : Incontinence management, prevention of pressure ulcers and promotion of self-care. | Geriatric assessment upon admission. Multidisciplinary meeting once a week (with the chief physician, resident, therapists, and a social worker); daily information, education and active participation by family or caregiver encouraged | Not reported |
| Saltvedt et al [21, 22] | Randomised controlled trial | 9 beds. | No | Ward | Patients were screened for eligibility by a research nurse. Screening only occurred in the presence of bed capacity on the AFU. States "Eligible patients who had been recently admitted to the department". Patients were allocated to the ward on the day of recruitment. | > 75 | At least one target condition from a list of 15 clinical and functional criteria  Exclusion  Acute stroke Care home residents  Predicted mortality < 6 months Metastatic cancer Expected LOS < 3 days | Mean age 81;  Mean LOS period to transfer to AFU 2 days (1-5).  Heart disease (36%) Infectious disease (25%) GI disturbance (21%) | Geriatrician led. Supported by a resident. Comparable nurse to bed ratio as general medical ward. Physiotherapist and OT. A SW could be consulted when necessary. | MDT twice a week to report assessments, set goals, discuss problems, and plan discharge. Early mobilization, with encouragement to participate in activities of daily living (ADLs) and communal meals was instituted to avoid further functional decline. | Frequency of assessment not reported at the patient level | During the study period 1426 patients over the age of 75 were admitted as emergencies to internal medicine. A total of 254 patients were randomised. The total number screened for eligibility is not reported. |
| Schubert et al [36] | Observational cohort | Mobile  Consultative service to admitting team | Na | ED   Ward | Screening of all patients >65 within 48 hours of admission to hospital. When the review was positive, team would contact the admitting team and offer to consult; the admitting team could accept or decline. Could also attend at admitting teams request | > 65 | Screening process looked for the presence of functional impairment, geriatric syndromes and frequent ED admission. | Mean age; 82  Common diagnostic list provided without proportions | Geriatrician led. Responsibility for patient remained with admitting team. | Provided CGA and supported parent team to implement plan. No further description on how this was operationalised. | Not reported | 793 patients were eligible for assessment. 421 received assessment. In 297 cases admitting team declined assessment and 75 cases team had no capacity. |
| Shaw et al [44] | Observational cohort  (no comparator) | 21 bed unit.   Adjacent 42-bed sub-acute medical (SAM) unit for prolonged convalescence. | Not reported | ED | Eligible patients who present to ED will be referred to the one of the following for admission to ACE: IMD specialist (CTU) on call for the day, the patient’s family physician, or the hospitalist for unattached patients. | >75 | "Age used as a convince proxy for frailty. Presence and/or potential of functional decline". | Not reported | Collaborative model of physician practice. Eligible patients could be under the care of both family practitioners or internal medicine specialist physicians  Geriatrician led. Responsibility for patient remained with admitting team. Access to physiotherapy, OT, SW, | Screening for delirium and incontinence on admission. | Daily round. Twice-weekly inter disciplinary rounds by a geriatrician. All patients were referred for a therapy assessment within 24 to 48 hours of hospital admission. | In the absence of available beds, some eligible patients are admitted to other wards as off-service patients. Formed a small proportion of total. |
| Taylor et al [45] | Observational cohot  (no comparator) | 12 bed   Located within an existing acute medical ward (MAU). | No | Ward after admission from ED. | Screened after referral to medicine. Older patients could also be referred and assessed by treating clinician. | > 75 | Functional and clinical criteria: falls, delirium, dementia or care home/intermediate care residents | Mean age; 85  Falls 32%  Delirium 20% | Geriatrician led, acute physician, nurse, physiotherapist, OT, SW, mental health liaison nurse and pharmacist | Not reported. Aim to faciliate same day discharge, Patients that could not be discharged some day transferred to specialist geriatric medicine ward. | Geriatrician-led CGA between Monday to Friday, 8:30am – 5pm | A geriatrician reviewed almost 50% of patients who met criteria for the COPE zone. CGA and MDT were undertaken 40.9% and 29.3% respectively. |
| Stewart et al [37] | Observational cohot | 12 beds. | No | ED | Allocated to the study groups based on bed availability. A nurse supervisor and the admitting house officer, who were not involved in the study determined eligibility | >85  or >75 with additional criteria | > 75 in the presence of a series of written clinical criteria related to geriatric syndromes and functional impairment | Mean age; 86 | Geriatrician led. Physician's assistant. Nurses with geriatric specialist training. | Not reported | Not reported | Not reported |
| Wald et al [38] | Non-randomised controlled trial | 12 beds. | No | Not reported | Not reported. Accepted patients between 0700 and 1500 Monday–Friday; between 0700 and 1200 Saturday and Sunday. | > 70 | No other selection criteria applied related to frailty. | Mean age; 81.  28% admitted with "pulmonary diagnosis" | Physician led (General internal medicine /hospitalist) with additional geriatric training. Physicians rotated to other hospitalist services throughout the academic year, including the usual care services. A novel geriatric educational curriculum was delivered to medicine residents and medical students. The nursing staff had no formal geriatric nursing training. | A brief, standardized geriatric assessment including screens of function, cognition, and mood; a clinical focus on mitigating the hazards of hospitalization, early discharge planning; and   The brief standardized geriatric assessment consisted of six validated instruments, and was completed by house staff or medical students on admission | 15 minute daily (Monday–Friday) multi-disciplinary rounds   Described and compared processes of care such as recognition and treatment of delirium | 217 patients met the eligibility criteria. 122 were admitted to the unit and 95 received usual care. Details or randomisation and allocation unclear. |
| Webb et al [46] | Observational cohort  (no comparator) | Not reported  Located in community hospital | Not reported | GP  Ambulance. | GPs would call the EMU when they were concerned about a patient, and felt a higher level of assessment, but were not acute enough to require ED. The exact process by which this was determined is not reported.   Access was limited to selected GPs. Accept referrals Monday-Friday. 0900-1700 | None | Accepted all adult emergencies except stroke and chest pain. | Not reported | Not reported | "Full tailored assessment" on presentation including X-ray, electrocardiogram, near-site blood testing and urinalysis. | Not reported | Not reported |
| Ribbnick et al [23] | Randomised controlled trial (pilot) | 23 beds   Located within an intermediate care facility. | Yes  Single rooms designed to accommodate respite for the informal caregivers. | ED | Triaged by geriatrician. Assessed appropriateness and consent for transfer to unit. | None | Presence of geriatric syndrome. | Not reported | Geriatrician led. Physician's assistant. Nurses with geriatric specialist training. Nurses had experience in hospital and community settings. | CGA on admission to unit. Focused on early mobilisation and rehabilitation. Physiotherapy and routine laboratory testing are available during the workweek and simple X-ray is available once a week. | Not reported | Pilot study |
| Wald et al [38] | Non-randomised controlled trial | 12 beds. | No | Not reported | Not reported. Accepted patients between 0700 and 1500 Monday–Friday; between 0700 and 1200 Saturday and Sunday. | > 70 | No other selection criteria applied related to frailty. | Mean age; 81.  28% admitted with "pulmonary diagnosis" | Physician led (General internal medicine /hospitalist) with additional geriatric training. Physicians rotated to other hospitalist services throughout the academic year, including the usual care services. A novel geriatric educational curriculum was delivered to medicine residents and medical students. The nursing staff had no formal geriatric nursing training. | A brief, standardized geriatric assessment including screens of function, cognition, and mood; a clinical focus on mitigating the hazards of hospitalization, early discharge planning; and   The brief standardized geriatric assessment consisted of six validated instruments, and was completed by house staff or medical students on admission | 15 minutes daily (Monday–Friday) multi-disciplinary rounds   Described and compared processes of care such as recognition and treatment of delirium | 217 patients met the eligibility criteria. 122 were admitted to the unit and 95 received usual care. Details or randomisation and allocation unclear. |

Table 2B

Hospital at Home care models

| **Author** | **Study type** | **Source of referral** | **Specific condition** | **Process of referral** | **Eligibility; Age** | **Eligibility; Clinical** | **Characteristics of included patients** | **Who: Team** | **Clinical governance** | **Treatment and diagnostics** | **Telehealth enabled** | **Out of hours arrangements** | **Standard of care** | **Frequency of assessment and intervention (as achieved)** | **Proportion of eligible patients assessed** |
| --- | --- | --- | --- | --- | --- | --- | --- | --- | --- | --- | --- | --- | --- | --- | --- |
| Aimonino Ricauda et al [47] | Randomised controlled trial | ED GP Ward | COPD | Patients were accepted after a after period of stability in ED between 12-24 hours.  Referral hours   Admits new patients 7 days a week. Hours not reported. | > 75 | Acute exacerbation of COPD  Exclusion    Severe hypoxemia  Severe acidosis or alkalosis  Pulmonary embolism;  Myocardial infarction; Severe renal impairment ,  Cancer,  Hepatic failure,  Severe dementia (Mini-Mental State Examination score <14) | Mean age; 80 | Geriatrician led. Nurses, physiotherapy, SW, counsellor | "From an administrative, legal, and financial standpoint, hospital-at-home patients were considered hospital inpatients until discharge from hospital-at-home care". | Range of hospital level diagnostic and treatment modalities. blood tests, electrocardiogram, spirometry, pulse oximetry, oxygen and other respiratory therapies, intravenous fluids, antibiotics and other medicines, blood transfusions, surgical treatment of pressure ulcers, echocardiograms, and Doppler ultra- sonographies. | No | Hospital-at- home staff was available at all times for urgent home visits. | Assessment including bloods and radiology performed in ED prior to ambulance transfer home. In the first days after admission to HaH, physicians and nurses visited each patient at home daily. In the following days, a nurse saw the patients every day and the doctor saw them at intervals of 2 to 3 days. Number of attendances could be tailored depending on clinical condition. Capacity of 25 patients per day. | Frequency of assessment not reported. 77% received intravenous antibiotics, 58% oxygen therapy, 44% intravenous steroid. | 529 patients assessed for eligibility.   347 deemed ineligible and excluded (Out of geographical catchment = 148, severe co-morbid illness 80, required invasive moniroting n = 74, No social or family support)  182 eligible   n = 78 refused randomisation Reasons for refusal not reported |
| Cai et al [68] | Observational cohort | ED Community services OP clinic | Medical | Evaluated medically before entering the program to ensure that they could be treated at home. Details of evaluation not reported   Referral hours   Not reported | "Veterans" | No formal elibility criteria   "Targeted medically stable patients that required more intensive care than could be provided by existing community or OP services"   The most common conditions treated included congestive heart failure, chronic obstructive pulmonary disease, community-acquired pneumonia, diabetic foot ulcer, and complicated wound care  Exclusion   Care home residents | Mean age; 68  Diagnosis not reported | Physician (speciality not stated). SW. dietician, pharmacist. Access to physiotherapy and OT, SLT via separate community team | HaH physician. Further details not reported. | Access to blood tests and intravenous treatment (diuretics and antibiotics). | No | HaH physician available 24 hours a day for urgent or emergent visits. | Initial admission visit from the HaH physician to initiate appro- priate treatment, and then the HaH registered nurses assessed within 24 hours and daily thereafter during the acute phase of their illness. Daily physician oversight was provided at each HaH nurse visit by phone. | Frequency of assessment and number of patients receiving individual interventions not reported | Not reported |
| Broard et al [48]  Caplan et al [49, 50] | Randomised controlled trial | ED | Medical | Referred by parent team and subsequently assessed by HaH physician prior to inclusion. If any doubts about suitability a before randomisation. . Only accepted if deemed to require admission by the relevant parent team.   Referral hours  Not reported | "Targeted patients over 65 but accepted younger patients" | No formal eligibility criteria reported.   "Teams encouraged to refer patients with acute pneumonia, urinary tract infections, and cellulitis and sub acute infections, (endocarditis and osteomyelitis) infections requiring treatment with intravenous antibiotics, deep venous thrombosis (DVT), minor cerebrovascular accidents (not affecting mobility or swallowing), and cardiac failure"  Exclusion  Shock Required supplemental oxygen Judged too unwell by the study   No available caregiver; lived outside the local area; or if their home was unsuitable for home treatment  Lived outside geographical catchment | The majority of patients were treated for infection with parenteral antibiotics. Other diagnoses included congestive cardiac failure (4), acute back pain (2), faecal impaction (2), acute myocardial infarct (1), shingles (1), anaemia (1) and cerebrovascular accident (1). | Involved care by hospital physician with support from GP to deliver care at home. Access to allied health care professional is not reported. | Care delivered by GP with support from secondary care. If GPs declined to participate, the hospital provided medical support. Before the study, an educational program consisting of an evening lecture and a question-and- answer session. | Administration of parenteral antibiotics and other medications, and blood transfusions. Infections were generally treated with once-daily intravnous antibiotics such as ceftriaxone, gentamicin, or vancomycin | No | Not reported | To be included in study patients discharged within 24 of assessment in ED. Standard of care with reference to frequency of visits is not reported. | Not reported | Not reported |
| Augustine et al [66]  Federman et al [70]   Saenger et al [78] | Observational cohort | ED GP | Medical, list of specific conditions | HaH clinicians reviewed and completed a history and examination to determine eligibility in ED.  Referal hours  Weekdays (0800-1600) | > 18 | Initially, eligible admission diagnoses were acute exacerbations of asthma or chronic obstructive pulmonary disease, decompensated congestive heart failure, urinary tract infection,, community-acquired pneumonia, cellulitis of the lower extremities, deep venous thrombosis or pulmonary embolism, hypertensive urgency, hyperglycaemia, and dehydration.   Over time, the number of eligible conditions expanded to 19, representing 65 diagnosis-related groups (DRGs). Details of the exact diagnoses accepted not provided.  Required medical admission fee-for-service medicare Lived in safe environment  Within geographical catchment.   Exclusion  Clinically unstable, required cardiac monitoring or intensive care. Clinical instablity not specifically defined. | Mean age; 76.9  The 4 most frequent admission diagnoses were urinary tract infections, community-acquired pneumonia, cellulitis, and congestive heart failure. | Physician led (speciality not reported), nurses, therapist, SW. | HaH physician. Further details not reported. | Intravenous infusions (eg, antibiotics, diuretics, fluids for rehydration), wound care. Durable medical equipment, phlebotomy, and home radiography were provided as needed. | Yes  Video conference calling | HaH physicians were available 24 hours a day. Able to direct paramedics who were available for urgent evaluation at any time. | Nurses visited patients once or more a day to provide most of the care, and a physician or nursing specialist saw patients at least daily in person or via video call facilitated by the nurse. A social worker visited each patient at least once | Frequency of assessment and number of patients receiving individual interventions not reported at the patient level | The number of patients reviewed for eligibility was not reported. 460 patients with HaH-qualifying conditions were approached: 19 were ineligible because of clinical instability or concerns about the home environment, 146 refused,   295 (63.9%) were eligible and agreed to HaH care |
| Gonzalez Barcala et al [71] | Observational cohort | ED | Acute respiratory illness | HAH physicians assessed patient in ED.  Referral hours  Not reported | Not reported | Diagnosis of respiratory infection, pneumonia or exacerbation of chronic obstructive pulmonary disease.   Required intravenous treatment, artificial nutrition, oxygen therapy or aerosol therapy to manage acute condition .  Additional geographic and "willingness criterion" applied.   Exclusion  Hemodynamic instability. Respiratory rate <25 per minute. Heart rate > 110 beats/min. | Mean age; 75   Stratified for respiratory illness Specific diagnosis not reported | Physician (speciality not stated). Nurse. No other details reported. | HaH physician | Intravenous treatment, acute oxygen therapy, nebulised therapy. | No | HAH physicians and nurses can be contacted by telephone by their patients every day between 8 am. and 8 pm . No specific provision outside of these hours | Daily visits by physician and nurse, until the patient's progress allows transfer to a less intensive regimen with fewer visits by hospital staff. Eventually, only telephone contact is maintained. | Frequency of assessment and number of patients receiving individual interventions not reported at the patient level | Not reported |
| Leff et al [75] | Observational cohort | ED | Medical, specific list of conditions | ED or ambulatory site and his or her eligibility status for HH determined.   Referal hours  Not reported | Not reported | Requiring acute hospital admission for one of four target illnesses:  CAP Decompensated HF COPD Cellulitis   Excluded  Care home residents | Mean age;   53% were treated for CHF. | HaH physician,. Nurses, aides, and other ancillary staff provided by a home health agency. Access to other allied health professionals not reported | HaH physician | intravenous fluids, intravenous antibiotics and other medicines, oxygen and other respiratory therapies.   Diagnostic studies and therapeutics that could not be provided at home, such as computerized tomography, magnetic resonance imaging, endoscopy, were available via brief visits to the appropriate outpatient resource of the acute hospital | Yes  Lifeline device | HaH physician available 24 hours a day for urgent or emergent visits. | The patient had at least daily physician visists. The patient had direct nursing supervision for the initial portion of HaH admission. Duration of initial direct supervision dependent on the level of illness acuity, at least 24 hours in the "development" phase of the intervention. After the first 24 hours, a Lifeline device was placed in the home. | Patients treated at home experienced a mean of 20 & 3.1 hours of continuous nursing at the start of their care, and received a mean of 4.4 & 1.6 physician visits and 3.7 4.3 nursing visits  Frequency of assessment and number of patients receiving individual interventions not reported. Oxygen therapy administered in 77%. | 478 patients with target illness presented for hospital admission.   104 ineligible as lived outside the catchment area and/or were not insured by Medicare.   Of the remaining 374, 229 (61%)were ineligible for HH care based on previously described medical eligibility criteria.  One hundred forty five (39%) were eligible Of those eligible 17 subjects were enrolled in HH (12%of eligible),   122 were missed for enrolment (84% of eligible), and six (4% of eligible) subjects were eligible for HaH but declined to enrol. |
| Isaia et al [74] | Observational cohort | ED GP Ward | Medical illness, high risk of delirium | Patients were first assessed in the ED with a baseline standard clinical evaluation: After ED evaluation, patients were transferred home.  Referral hours  Not reported | > 75 | Study focused on the provision of HaH care at high risk of delirium but without evidence of delirium on presentation defined by CAM criteria.   How the HaH study inclusion criteria differred froim the normal inclusion criteria for the HaH service is not reported.   Exclusion   Care home residents Severe dementia Terminal illness | Mean age; 86.1  Diagnosis not reported | MDT consisting of three geriatricians, 13 nurses, two physiotherapists, one social worker and one counsellor | From an administrative, legal, and financial standpoint, hospital-at-home patients are considered hospital inpatients until discharge. | Blood tests, electrocardiogram, spirometry, pulse oximetry, oxygen and other respiratory therapies, intravenous fluids, antimicrobials and other medicines, blood transfusions, surgical treatment of pressure ulcers, echocardiograms, echographs, and Doppler ultra- sonographies. | No | Not reported | Not reported. | Frequency of assessment and number of patients receiving individual interventions not reported at the patient level | 2,930 patients aged 75 and older were consecutively admitted to the ED of our hospital for different acute illnesses.   Of these, 46.7% (n=1,370) needed hospital level care and 360 were admitted to the Geriatric Care Unit. Among them, 144 were eligible for this study:  Of the 216 patients excluded, 69 did not have a caregiver, 10 had a terminal illness, 53 were unable to undergo interviewing because of profound dementia (n = 37), coma (n = 6), aphasia (n = 4) or intubation (n = 4). The remaining 84 eligible patients refused to give consent. |
| Burton et al [67]   Clark et al [69]  Greenough et al [72, 73]  Leff et al [76]  Marstellar et al [77] | Non-randomised controlled trial | ED  Ambulatory site | Medical  Specific list of conditions; provided in supporting reference (X) | Patients were identified in an ED or at an ambulatory site at times when Hospital at Home could admit patients    Referal hours  Daily (0600-2200) | >65 | Required an acute hospital admission for one of four target illnesses: community-acquired pneumo- nia (CAP),an exacerbation of chronic heart failure (CHF),an exacerbation of chronic obstructive airways disease (COAD), or cellulitis; and met previously described medical eligibility criteria for HH.   Exclusion   Care home residents | Mean age; 76.2  CAP 54% COPD 32% CHF (25%) Cellulitis 12% | HaH physician, speciality not reported. Nurses. | 3 Medicare-managed care (Medicare Choice) health systems at 2 sites and a Veterans Administration medical center.  HaH physician   "The patient was followed by the same hospital-at-home physician until his or her condition was stable enough for discharge" | intravenous fluids, intravenous antibiotics and other medicines, oxygen and other respiratory therapies.   Diagnostic studies and therapeutics that could not be provided at home, such as computerized tomography, magnetic resonance imaging, endoscopy, were available via brief visits to the appropriate outpatient resource of the acute hospital | Yes  Lifeline device | HaH physician available 24 hours a day for urgent or emergent visits. | Patients were evaluated by the HaH physician either in the ED or shortly after arriving at home. The HaH nurse met the ambulance at the patient’s home.The patient had subsequent direct one-on-one nursing supervision for an initial period of at least 8 hours at site 3 and for a period of 24 hours at sites 1 and 2. HaH physician visited at least daily   When direct nursing supervision was no longer re- quired, the patient had intermittent nursing visits at least daily. The hospital-at-home physician made at least daily home visits | Frequency of assessment difficult to assess due to intention-to-treat analysis.   Patients in HaH group mean of 1.69 hours of continuous nursing at the start of their care, and received a mean of 1.5 physician visits and 1.4 nursing visits a day.   Number of patients receiving individual interventions were reported but analysis using intention to treat means difficult to determine how many recived these treatments at home. | Target sample of 985 patients , 771 ineligble as had an acute illness, other than the target illness requiring admission  169 offered HaH of which 84 received intervention. (77 declined HaH treatment and 73 not offered HaH treatment)  The most common reasons for medical ineligibility were un- correctable hypoxemia, suspected myocardial ischemia, and the presence of an acute illness that required hospital admission other than the target illness. |
| Levine et al [53, 54, 55] |  | ED | Medical, specific list of conditions | ED attending physician made decision to admit and call the triage hospitalist as per usual protocol.   If the triage hospitalist agreed that the patient met preliminary inclusion criteria, the HaH tea assessed the patient for eligibility  Referal hours  Not reported | > 18 | Primary diagnosis of any infection, heart failure exacerbation, chronic obstructive pulmonary disease exacerbation, asthma exacerbation, or selected other conditions within a written document.   Exclusion    Care home residents  High risk for clinical deterioration on the basis of validated general and disease-specific risk algorithms provided within study appendix (eg CURB65).  Required administration of controlled sub- stances Assistance of 1 person to reach a bedside commode | Mean age; 80  Diagnosis  Infection 53% (CAP / cellulitis) CHF 16% COPD 12% | Physician led by general internists. Training involved a 1-day didactic course and several days of shadowing physicians experienced in home medicine prior to Woking in the program. home health aides, physiotherapists, OT, SW. | HaH physician. Further details not reported. | Intravenous medications via infusion pump. Respiratory therapies (such as oxygen), in-home radiology. Point-of-care blood diagnostics using Abbott platform. | Yes  All patients had continuous monitoring of temperature, heart rate, respiratory rate, telemetry, movement, and falls via a small skin patch (VitalConnect). This monitoring was done through machine-based algorithms, which produced alarms for review by both nurse and physician (delivered to their smartphones). | The home hospital attending physician was available 24 hours a day for urgent issues and visits. | All patients received at least 1 daily visit from an attending general internist and 2 daily visits from a home health registered nurse, with additional visits as needed. The physician–patient ratios for home hospital were capped at 1:4. No specific treatment pathways or algorithms employed. | During HaH episode, home patients had less imaging (median per- centage of patients, 14% vs. 44%), had fewer laboratory orders (median per admission, 3 vs. 15 orders) | 43 allocated to HaH 248 assessed for eligibility,  157 excluded (patient declined n = 85, caregiver declined n = 36, ED physician declined = 24, OP physician declined n = 3 ) |
| Mader et al [81] |  | ED | Medical, specific list of conditions | Assessed by nurse. Electronic record review by physician before admission.   Referral hours    Daily (0800 -1630). | Service targeted at older patients but did not exclude younger patients. | The authors provide details of modifications a previously reported HaH model (Leff).   Exclusion   Care home residents | Mean age; 68  Diagnosis  CHF 51% COPD 19% Pneumonia 11% Cellulitis 25% | Physicians were general internists or family physicians with Certificates of Added Qualifications in Geriatrics experience in home care, nursing home care, and hospital care. Oth  Physiotherapy, OT, SW, pharmacy, respiratory therapists were accessed using "existing OP patient access mechanisms" | HaH physician. Further details not reported. | Access to home radiology and electrocardiogram were maintained, but they were available but rarely used. | No | Not reported. | Daily nursing visits Single physician visit, with daily physician oversight and additional physician visits if needed. Average daily census ranged from zero to three patients. | Frequency of assessment not reported  Approximately 50% of the patients received intravenous therapy, oxygen therapy, or both, although only 20% of the oxygen therapy was a new. Approximately 20% of the patients required some kind of wound care | Not reported. Average daily census ranged from zero to three patients. |
| Mas et al [84] | Observational cohort | ED Day hospital | Medical | Not reported  Referral hours  Not reported | Not reported | Older patients with "chronic conditions". Targeted patients as having a "good prognosis" and not needing further complex diagnostic tests or management  Exclusion   Lived alone | Mean age; 84  Diagnosis  Respiratory infection 42% CHF 28% UTI 19.3%  Functional impairment due to acute illness 56% Delirium 18% | involved care by hosptial physician with support from GP to deliver care at home. Access to allied health care professional is not reported. | HaH physician. Further details not reported. | Electrocardiography, blood tests, and imaging studies. Intravenous antibiotics, corticosteroids, diuretics and fluids, and nebulizers for bronchodilator therapies. | No | on-call physicians could be reached by phone 24 hours. Capacity for home visit OOH not stated directly. | All patients received an initial assessment and treatment visit from a nurse specialized in geriatrics within 12 hours of referral and an initial visit from a geriatrician within 24 hours of referral.  Patients in the HaH group received 1 to 3 individualized home visits from staff between 0800 and 2100 daily. Patients were visited by a physician daily or every other day, and by a nurse twice a day or daily | Frequency of assessment and number of patients receiving individual interventions not reported at the patient level | Not reported |
| Mendoza et al [57] | Randomised controlled trial | ED GP | Decompensated HF | Assessed by and referred by ED doctor. Diagnosed decompensation of CHF and initial eligibility screen. Reviewed by HaH team in ED before inclusion.   Referal hours    Admit new patients between the hours of 6 am and 8 pm daily | > 65 | Admitted in the preceding 2 months for deterioration of HF or acute coronary syndrome. NYHA II/III  Exclusion  Presence of severe symptoms  ACS | Mean age; 80 | Physician (general internal medicine). Nurse. | HaH physician. Further details not reported. | Electrocardiography, blood tests, intravenous diuretic | No | No specific provision | Patients assessed at home within 12 – 24 h of leaving ED. In the case of deterioration or no response to treatment, with transfer to the cardiology ward.  Patients were visited by a physician daily or every other day depending on their clinical condition. Visited daily by a specialist nurse. | Frequency of assessment and number of patients receiving individual interventions not reported at the patient level | Not reported |
| Dismore et al [51]  Echevarria et al [52] | Randomised controlled trial | Acute Medical Ward | Acute exacerbation of COPD | Not eligible from ED (to esnure only patients admitted to hospital included) process of case identification not reported | > 35 | Patients with COPD and acute exacerbation.  Low risk DECAF (0–1).   Exclusion  Coexistent secondary reason for admission  illness (other than COPD) likely to limit survival to less than 1 year long term ventilation, | Mean age; 71 | Respiratory physician’s specialist nurse. Physiotherapy, OT and formal social care were available at home. | Hospital respiratory team | Oral and intravenous therapies, acute controlled oxygen therapy were available. |  | Not reported | Admitted to hospital and assessed in ED. identified as low risk by DECAF, and then returned home under the care of the hospital respiratory team, usually within 24 hours of admission  Patients received once or twice daily visits from respiratory specialist nurse. | Including travel time, healthcare professionals spent a median of 7.2 hours (IQR 4.7–10.8) on home visits per HAH spell (median RSN visits=7.1hours, IQR 4.4–10.1) | 207 patients assessed eligibility.  87 excluded (Not meeting inclusion criteria n = 50, decline to participate n = 37)  58 allocated to HaH. All received the intended intervention |
| Montalto et al [82] | Observational cohort (No comparator) | ED Ward | Medical | Not reported | > 18 | Treatment requires hospitalisation no other specific criteria provided | Mean age not reported   Majority treated for infection Soft tissue infection 32% DVT 14% | Physycian led. Specialist nurses. Access to allied health professional not reported | "Retained by hospital" | Intravenous antibiotics, fluid, blood transfusion | No | 24 hour cover by HaH team | Not reported. | Mean of 4.1 visits per admission (s.d. = 2.7); and 31 751 nursing visits (mean = 9.3 per admission, s.d. = 9.8) were delivered. | 3423 admissions over a 7 year period. Proportion potentially eligble not reported. |
| Patel et al [58] | Randomised controlled trial | ED Medical ward OP clinic | Decompensated HF | Identified within 48 hours of presentation. Assessed by cardiologist prior to enrolment. Process of case screening for eligbility not described. | No age restriction | Clinical evidence of decompensated HF Known HF  Exclusion  New onset HF Pulmonary oedema  Symptoms < 3 days Care home resident  Trop > 0.05 Creatinine > 250 | Mean age; 77 | Cardiologist. Specialist HF nurse. | Hospital cardiologist | Intravenous diuretics | No | Not reported | All patients were followed-up the day after returning home by a specialist nurse from the HF clinic. Visited at home daily or every other day by the specialist nurse for 5–7 day. | Average number of home visits 4 (3.5-4). | 1127 patients screened, 786 potentially eligible of which 755 were excluded (116 declined to participate). 31 randomised of which 13 received HaH care. |
| Pouw et al [64] | Protocol | ED | Medical illness with co-existent cognitive impairment (dementia or delirium) | Patients identified in ED  Referral hours  Recruited within "Office hours" | > 65 | Required acute medical admission in opinion of ED.  MEWS < 3 Informal care giver   Exclusion   Care home residents Hospitalised previous 7 days Expected terminal event | Protocol: not reported | Physician (speciality not defined), nurse, pharmacist and physiotherapist. Other allied health care professionals (eg, a dietician, occupational therapist or social services) could be involved on request. | "The HaH team works under the responsibility of the medical specialist in the hospital" | Intravenous therapy (eg, antibiotics, fluid, and/or diuretics), oxygen therapy, and/or nebuliser, indwelling urine catheter or a nasopharyngeal food tube. Diagnostic procedures and therapeutics that cannot wait and are not available at home, such as endoscopy or CT scan, will be arranged through brief visits to the hospital. | Yes  Medical alert device in the house, with a 24/7 connection to an on-call service. | The Hospital at Home physician and nurse will be available for emergency visits. Whether this relates to OOH provision is not made explicit. | The HaH nurse is responsible for the day-to-day care and will be present on arrival of the participant at his/her residence.   After the care intake and a period of direct nursing supervision, the participant will receive intermittent nursing visits daily (starting with three times per day), including weekends and public holidays. The Hospital at Home physician will make a home visit every day (excluding weekends) | Protocol: not reported | Protocol: not reported |
| Salazar et al [83] | Observational cohort (No comparator) | ED Ward | Not reported | Not reported | Not reported | No specific clinical criteria reported Home carer available ("no social problems") "Diagnosis confirmed in ED" but no further details provided | Mean age; 75  COPD 50.8% CHF 12.8% CAP 9.6% UTI 8% DVT 6% | Two attending doctors and two nurses are assigned specifically to the unit. Speciality of physicians not reported. | Not reported | Access to laboratory and electrocardiography testing. Can provide acute home oxygen. | No | on-call physicians could be reached by phone 24 hours. Capacity for home visit OOH not stated directly. | Not reported. | The frequency of patient assessment and specific interventions not reported at the patient level. | Not reported |
| Sheppered et al [59, 65]  Mäkelä et al [56] | Randomised controlled trial | ED AMU ( dedicated unit for management of acute medical emergencies) GP Ambulance (1 unit) | Medical | Participants were referred to the trial by ED or GP if recruited from home. admissions to the MAU were also screened for eligible participants. Assessed by a nurse practitioner within one to four hours of referral and discussed with a consultant prior to inclusion   Referal hours  Not reported | >65 | Referred to a geriatrician led HaH led service with ability to perform CGA. Patients considered too "high risk" for home treatment were excluded at the discretion of HaH physician. Clinical criteria in relation to the safe spectrum of acuity are not reported.   Exclusion  Unsafe home evironment  Care home resident  ACS  Stroke  EOLC | Mean age; 83  Presenting syndrome  Acute fucntional decline 37% Fall 21% Respiratory tract infection 5% CHF 2%   Diagnosis  Infection 45% Cardiovascular diagnosis 13% | Geriatrician and specialty-training registrars in (7 of 9 sites recruiting 90% of patients)) GP clinically responsible in 2 sites. Included nurse practitioners who shared responsibility with the geriatrician for clinical assessments. Access to physiotherapists; OT, SW, mental health nurses and old age psychiatry | In all but one site the CGA HAH services have clinical governance arrangements with the acute hospital directorates | In all but two sites IV antibiotics could be administered. In all but one site nurses can administer IV fluids. Most services provided home oxygen. ECG, monitoring, urinary catheterization, post-void bladder scanning, dressings to skin lesions. | No | All sites provided health care in the evening, up to 8pm or 11pm. All sites had access to the usual NHS telephone consultation out-of-hours service. No specific arrangements. | Frequency of face-to-face assessment not specifically stated. CGA geriatrician led and occurred prior to discharge from hospital or at home within 24 hours. Care discussed at daily board rounds. In all sites there was at board round at least a daily (Monday to Friday), a review at the end of the day could also occur   Patients admitted to HaH directly from home assessed by a senior nurse, and an attending geriatrician if they have complex health problems within 24 hours.   . | The frequency of assessment and proportion receiving individual interventions not reported. | 1055 patients randomised  700 assigned to HaH of which 659 received HaH care  355 assigned to usual treatment of which 76 ultimately received HaH treatment |
| Skjot-Arkil et al [60] | Randomised controlled trial | ED | Medical | Referral to the study was made through the GPs in their weekday opening hours the patient or the municipal nurse on the patient’s behalf concerning an acute medical problem. f the GP found, that the patient was in a condition that would require acute hospital in-patient care the patient was eligible for inclusion in the study  Referal hours   Not reported | 65 | Acute hospital in-patient care deemed required by the GP. Use of a triage score but this did not dictate eligibility. Level of acuity difficult to determine.  Exclusion  Not reported | Mean 83  Diagnosis  Pneumonia 30% COPD 17% Dehydration 17% Delirium 8%  Triage   Green 54% Yellow 25% Orange 16% Red 5% | An optional training programme in basic HaH service was offered to the all participating GP. 94 GPs participated. 8 physicians, all specialists in internal medicine, participated in the study and they all received similar training. For both groups, the HaH nursing care was performed by the municipally employed nurses | Trial to investigate the difference between HaH care models with GP or ED physicians as clinician responsible for care  The GP responsible for the treatment prescribed mediations and could be contacted by telephone during daytime hours. Hospital specialists could be contacted directly by telephone within a 48 h period after initial assessment but were unable to provide home visits. | Intravenous antibiotics and fluids. | No | Outside working hours, the local GP on duty could be consulted | Processes not reported in detail beyond initial assessment. HaH nurses could visit up to 8 times per day. . | Frequency of assessment and interventions delivered not reported at the patient level | 139 referred from GP 8 did not meet eligbility criteria  Number of potentially eligible patients not reported |
| Tibaldi et al [61] | Randomised controlled trial | ED, GP Ward | Dementia and acute medical illness | Not reported   The appropriateness of HaH treatment was at the discretion of the HaH team.  Referral hours   Daily (12 hours) | No | Accepted patients with undifferentiated medical conditions needing hospitalization but not expected to require emergency interventions  Exclusion   No appropriate care supervision No telephone connection  Outside geographical catchment | Mean age; 83  Infection 35% Malnutrition 17% ). | Geriatrician led. Team contained 5 geriatricians, 14 nurses, 4 physiotherapists, 1 dietician, 1 social worker, 1 counsellor. | "HaH patients are considered hospital patients, and all services are provided by the hospital, which retains legal and financial responsibility for care." | Not reported but previous description of same model report access to a range of intravenous treatments. | No | The home hospital attending physicians and nurses contactable 24 hours a day. Unclear if this also includes the capability to provide urgent face-to-face review out of hours. | Not reported | The frequency of assessment and proportion receiving individual interventions not reported. | Not reported |
| Tibaldi et al [62] | Randomised controlled trial | ED Ward OP clinic / community | Decompensated HF | All patients admitted to ED were screened for potential elibibility.  Referral hours   Not reported | 75 | CHF and a persistent functional impairment indicative of New York Heart Association (NYHA) class III or IV stat when Deemed to require admission from ED, need for intravenous drug infusion  Exclusion criteria  new-onset heart failure;  absence of family and social support;  need for mechanical ventilation,  hemodialysis, or intensive monitoring;  severe dementia | Mean age 82 | Geriatrician led. 4 geriatricians, 13 nurses, 3 physiotherapists, 1 social worker, and 1 counsellor. | HaH patients are considered hospital patients, and all services are provided by the hospital, which retains legal and financial responsibility for care. | Blood tests, pulse oximetry, spirometry, electrocardiography, echocardiography, doppler ultrasonography, ambulatory electrocardiography and arterial blood pressure monitoring, oral and intravenous medication administration (such as antibiotics and cytotoxic drugs), oxygen therapy, blood products transfusion, central venous access, surgical treatment of pressure sores | No | The home hospital attending physicians and nurses contactable 24 hours a day. Unclear if this also includes the capability to provide urgent face-to-face review out of hours. | Initially patients were visited at home on a daily basis by physicians and nurses. In the following days the patients were seen by a nurse and the physician at intervals of 2 to 3 days or less, as required by the clinical condition of the patient. | The frequency of assessment and proportion receiving individual interventions not reported. | 528 patients assessed 342 ineligible (out of catchment n = 150, severe comorbidity n =78, intensive monitoring n = 59, no social support n = 55)  186 potentially eligible  85 refused   48 randomised to HaH care |
| Tsiachristas et al [78] | Observational cohort | Not reported | Medical | Not reported | 65 | Exclusion  Surgical emergencies Stroke ACS | Mean age 81 | Geriatrician led. Advanced nurse specilaists. | Not reported | Diagnostics such as radiology, and intravenous fluids, antibiotics and oxygen. | No | Standard primary care provision | Initially patients reviewed in own home. Daily MDT meetings to discuss care. | Not reported | Not reported |

Table 2C

ED in reach care models

| **Author** | **Study type** | **Model** | **Where:** | **Environmental adaptations** | **Source of referral** | **Eligibility; Age** | **Eligibility; Clinical** | **Process of referral** | **Population** | **Who: Team and training** | **Standard of care: work as imagined** | **Frequency of assessment: work as done** | **Proportion of eligible patients assessed** |
| --- | --- | --- | --- | --- | --- | --- | --- | --- | --- | --- | --- | --- | --- |
| Alakare et al [84] | Randomised controlled trial | Geriatrician in ED | No bedded capacity | NA | ED | > 75 | Clinical Frailty Score (CFS) 4 or more  Exclusion  Not reported | Screening for eligibility by ED secretary that assigned ensured met the age and residency criteria. ED nurses. Nurses assessed CFS. Patient enrolment was active" during office hours" | Median age; 85  Diagnosis not reported Median CFS 5-6 | Two geriatricians and two emergency physicians with consultation support from a geriatrician provided the assessment.   A Geriatric nurse and physical therapist from community hospital helped to organise support, care, and at-home rehabilitation for discharged patient | Received CGA by geriatrician. Included evaluation of function and impact on activities of daily living and observed the patients’ ability to walk.  The Abbreviated Mental Test 4 (AMT4) and Six-Item-Screener were used for assessment of cognitive status   ED team physician was in charge of the acute care for all enrolled patients, the geriatric assessment was led by second physician | The study physicians gave and documented at least one recommendation for 202/213 (94.8 %) patients. The most frequent recommendation was about medication (given to 168/213; 78.9 % of the patients), followed by advice on organising home care or rehabilitation-at- home services (65/213; 30.5 %) | 4356 eligible patients by age criteria. CFS not assessed in 1968 (45%). CFS 4-9 as assessed by nurse n = 1711 of which 506 presented during operating hours. 441 were randomised of which 215 received the intervention. |
| Ardents et al [87] | Before and after (uncontrolled) | AFU within ED CDU. | 2 beds. | Yes   Low level bed and direct visualisation from nursing station | ED | Not reported | Not reported   Exclusion  Australasian Triage Scale 1 (resuscitation) or 2 (emergency) Suspected fractured hip Suspected stroke,  a conscious state after fall that was different to baseline for the patient, fall had occurred >48 h before presentation. | Patients were identified at triage and moved directly to AFU attached to ED. Process of patient identification at triage no reported. | Median age; 83  Diagnosis not reported | Geriatrician review of high risk patient sub-group. Trained hospital volunteers used orientation strategies. | Standardised four-point assessment of in addition to a normal ED assessment, including cognitive screening with the 4AT, pharmacy review of high-risk medications, discharge risk assessment using the Triage Risk Screening Tool (TRST).   Unclear who undertook the assessment. Daily geriatrician led ward round of high risk patients (TRST score > 2) | Not reported | Not reported |
| Argento et al [99] | Observational cohort (no comparator) | Nurse led ED care coordination | No bedded capacity. | NA | ED | > 65 | Triage Risk Screening Tool (TRST) 2 or more   Exclusion  Not reported | Nurse contacted by ED team directly. Could also be identified without prior referral. All over 65s screened. If TRST > 2 nurse contacts ED physician to determine appropriateness of assessment. Assess patient 7 days a week (0900-1900) | Not reported | Advanced nurse practitioner. | Perform CGA in ED. Includes Folstein Mini Mental Status Examination (MMSE), Confusion Assessment Method (CAM), and a Geriatric Depression Screening (GDS). Clinical ownership of patients remains with ED team. | Not reported | 1302 patients assessed with a 12 month period. Average of 5 assessments a day. This represented 12% of all the patients over 65 presenting to ED. |
| Basic et al [85] | Randomised controlled trial | Nurse led ED care coordination | No bedded capacity. | NA | ED | None | "Targeted those with functional or psychological dependence".   Exclusion   "severely ill". No specific details on how this was defined. | Not reported | Mean age; 79  Diagnostic groups reported   Cardiovascular (20%) Neurological (14%) Musculoskeletal (27%) Infection (14%) | Two specialist nurses. Nature of training not reported. | Initial assessment in the ED by a specialist nurse. Range of validated tools used to determine functional impairment, psychological and cognitive impairment. | Not reported | 795 patients referred for assessment of which 408 (51%) deemed eligible. 244 were randomised. Reasons for non-randomisation not reported |
| Buttery et al [88] | Before and after (uncontrolled) | Geriatrician in ED | No bedded capacity. | NA | All medical admissions | 70 | Aged >70.   Exclusion  Not reported | All medical admissions were screened. Used a "CGA tool to identify moderate-high clinical risk" within 24 hours of admission on weekdays. | Mean age; 83  Not reported | Geriatrician led. elderly care nurse specialist, elderly care specialist physiotherapist. | Patients were discussed or reviewed by the geriatrician, and depending on clinical need, actions were: (i) rapid transfer to geriatric wards (ii) case management on general medicine wards or (iii) facilitated discharge with referrals to appropriate geriatric clinics. | Reported proportion of patients with specific problems identified by the specialist team | Not reported |
| Clarfield et al [100] | Observational cohort (no comparator) | Geriatrician in ED | No bedded capacity. | NA | ED | Not reported | No written eligibility criteria | Patients are screened by a triage nurse in the ED  ED staff request a geriatric consultation.  Typically patents with complex multiple problems were referred.  The geriatric nurse also screens admission by attending a morning round in ED    Operational 7 days a s week (0800 to 1700) |  | Geriaction led. Geriatrician. Full-time nurse clinician, and part-time physiotherapist and OT. A SW attached to the ED works in close conjunction with the team. | Geriaction review in ED. Process not otherwise described, | Not reported | Not reported |
| Conroy et al [89] | Before and after (uncontrolled) | AFU within ED CDU. | 16 beds. | Not reported | ED | None | Not reported   Exclusion  Not reported | Patients admitted to a CDU of the ED. Referral to CDU at discretion of ED. Selected for patients deemed likely to be discharged within 24 hours. No specific selection criteria or referral pathways described. Geriatrician also provided in-reach into ED to identify suitable patients. | Not reported | Embedded geriatrician between 8-6pm 7 days a week. Community matrons to arrange POC and coordinate services across care interface. | A standardised integrated proforma was developed, along with care pathways guiding the care of frail older people within the main ED. In addition to a daily ward | Not reported | Not reported |
| Ellis et al [90] | Before and after (uncontrolled) | AFU within ED CDU. | 4 bes. | Not reported | ED | > 65 | Functional impairment  Cognitive impairment  Geriatric syndrome Fall NH resident   Exclusion  Functional independent  Single organ pathology requiring specialist input | Patients deemed by ED to require admission. The process by which patients were selected to for the observation unit as opposed to a general medical bed is not described. | Mean age; 81  Diagnosis not reported | Not reported   Same access to early supported discharge services and urgent associated health professional a assessment as patients in the medical receiving unit. | Patients were intended to receive CGA and either admission avoidance where appropriate or direct specialty admission.   Patients kept in their own clothes if at all possible. Al prescribing was reviewed by a consultant and medicines reconciliation conducted with a view to reducing polypharmacy. | Not reported | Not reported |
| Elias et al [111] | Observational cohort (no comparator) | SDEC unit | 6 assessment cubicles and two rooms located within a community hospital | Not reported | GP  Ambulance | None | No specific written criteria  Exclusion  None | Direct referral to senior clinician or nurse. Could not self-present to the service. No absolute exclusion criteria. Patients with high degree of acuity without an advance care plan redirected to acute hospital. | Mean age; 75  Diagnosis not reported | Led by Geriatrician or GP. Nurse specialists. Physiotherapist OT, SW. | Referred for assessment in EDU. Assessed using structured proforma containing validated tools for delirium. Point of care blood tests obtained. X-ray on-site. Additional imaging by acute hospital as required.  After assessment could be discharge, referred to HaH team or admitted to acute or community hospital bed. A dedicated transport service was available if required. | Not reported | 533 new assessment reported within a 4 month period. 152 patients admitted directly to acute medical bed. 58 were subsequently admitted to hospital in next 30 days. 315 were ultimately managed on an ambulatory pathway. |
| Foo et al [91] | Before and after (uncontrolled) | AFU within ED CDU. | 24 beds on CDU. Number of AFU dedicated beds not reported. | Not reported | ED | 65 | Not reported   Exclusion   Nursing home residents, poor premorbid cognition or function (e.g. advanced dementia, bed-bound) already under the care of a geriatrician | Process not reported. Unclear how patients selected for assessment. Appears to be triaged by nurse on the unit Appears to be at the discretion of the nurse providing assessment | Mean age; 77  Head injury 52% Hypoglycaemia 20% Cellulitis 9% | ED nurse with additional geriatric training. Nature of training not reported. | An ED nurse trained in geriatric care was located in the ED CDU. Provided geriatric assessment. The nurse discussed each case with either an ED physician trained in geriatric care, or a geriatric nurse clinician. Assessment included Timed up and Go Test. Physiotherapist referral if indicated,  Complex cases were referred onwards to geriatric outpatient clinic. Geriatric nurse clinician discussed complex cases at biweekly MDT meetings attended by a geriatrician | A total of 28% of patients assessed did not require any intervention by the service. Some 19.7% required but refused recommended treatment plan. | 621 patients presented to ED during the study period and met inclusion criteria. 315 (51%) received the intervention, 151 (25%) were "missed". The reason for exclusion not reported in 17% |
| Fox et al [101] | Observational cohort (no comparator) | Geriatrician in ED | No bedded capacity | NA | ED | None | Targeted patients "from residential care or intermediate care, presenting with confusion as a result of dementia or delirium or admitted with a fall"  Exclusion  Not reported | Patients were reviewed either directly after triage, after assessment by the ED team or after assessment by a junior doctor or advanced nurse practitioner (ANP) from the medical team. | Mean age; 85  Diagnosis not reported | A consultant geriatrician was based in the ED between 10 a.m. and 8 p.m. 7-day/week. The service did not extend into the night, when medical cover was delivered by the on-call service.  MDT comprising of nursing staff, , physiotherapist OT and SW. | Every patient underwent geriatrician-led CGA in ED. An all-inclusive CGA document was created for each patient clearly outlining functional and medical baselines, progress, problems and the plan of care. After assessment, patients were either discharged home or to intermediate care, or admitted to the AMU or directly to an Ageing and Complex Medicine (ACM) ward. | Not reported | 168 assessed in ED. Number potentially eligible not reported. |
| Gentric et al [102] | Observational cohort (no comparator) | Geriatrician in ED | No bedded capacity | NA | ED | 75 | No specific written criteria   Exclusion  Need for rapid diagnostic or therapeutic treatment (eg myocardial infarction) | Not reported | Mean age; 86 | Geriatrician led. Two SW, | Evaluation within a designated area of the ED. Evaluation focused on functional impairment, gait, balance and cognitive impairment. A "social survey" conducted by the SW. | Not reported | 1514 patients were assessed during a two year study period. This represented approximately 30% of patients over 75 presenting to the ED within the study period. |
| Hwang et al [92] | Observational cohort | Nurse led ED care coordination | No bedded capacity. | NA | ED | None | Identification of Senior At Risk score (ISAR) scores ≥4,  Emergency Severity Index (ESI) ≥3,  hospital discharge 30 days prior to the index ED visit | Referred directly by ED team. Used specific eligibility criteria but unclear whether patients were routinely screened or a referral required. Assessed patients weekday (0800-1600) | Not reported | Specialty trained ED nurses with a minimum of 5 years experience and additional training in geriatric medicine. | ED team retained clinical ownership of patient. Team nurses facilitate care transitions of older adults in the ED to the community with the goal of avoiding inpatient admission. Multiple validated tools were used to assess for functional status, cognitive impairment and delirium. | Not reported | 2137 of 21,923 (10%) of potentially eligible patients received the intervention. |
| Jones et al [103] | Observational cohort (no comparator) | Geriatrician in ED | No bedded capacity. | NA | ED | 65 | No specific written criteria   Exclusion  Not reported | Patients assessed at geriatricians discretion. No formalised criteria. Process of selection and need for prior assessment by ED or medical team not reported. | Not reported | Geriatrician led. 5.5 clinical sessions per week based in the ED. Physiotherapist provided by a "REACT" team. | Clinical processes not reported in detail. The geriatrician provided an elderly care clinic with multidisciplinary support in the medical day hospital for patients who were discharged from the ED, on a ‘rapid access’ basis where necessary. | Not reported | 848 assessed in ED during a 2 month period. Number potentially eligible not reported. |
| Khan et al [104] | Observational cohort (no comparator) | AFU within ED CDU | 8 beds. | No "The unit was not specifically designed for the care of older patients" | ED. | 65 | No specific written criteria   Exclusion  Not reported | Patients were referred to a CDU on the ED at the discretion of the ED team. Patients were referred to the unit if ED team felt likely "to need only a brief period of treatment or observation (less than. 24 hours). How this decision was made is not reported. | Mean age not reported  "Commonest reason for admission fall or poor mobility" | Care provided by the ED team. Assessed by an SHO and registrar where appropriate. Geriatricians not involved. Dedicated physiotherapist, OT and SW. Therapists operated Monday to Friday. | Not reported | Not reported | Not reported |
| Kwon et al [93] | Observational cohort | Nurse led ED care coordination | No bedded capacity. | NA | ED | 65 | Patients deemed "likely" to be discharged home at the discretion of the ED team. A least one prior ED visit within the last year, | The team screened all admissions using a tracking board. | Mean age not reported  Falls 34% Pain 19% | Geriatric and Palliative Medicine physician, an ED physician,   SW specialized in Geriatrics and Palliative Medicine and administrative assitant | The frailty team provided care coordination while the ED team provided clinical care. Consultation by Geriatric and Palliative Medicine physicians available as needed. Coordinated links to community resources, and conversations on advanced care planning. | Not reported | 535 patients assessed of which 283 deemed eligble. |
| Leung et al [94] | Observational cohort | AFU within ED CDU | 15 beds | Not reported | ED | 65 | No specific written criteria   Exclusion  Care home residents. | Not reported | Mean age; 84  Diagnosis not reported | ED physician led. Supported by geriatrician. Nurse case managed, Physiotherapist, OT | The processes of care are not well defined. Within 24h of admission, a consultant or specialist in emergency medicine will conducted a daily assessment with a geriatrician, nurse case manager, physiotherapist and OT. Treatment care plan will be developed in a multi-disciplinary approach but clinical ownership remains with ED physician. | Not reported | Not reported |
| Lo et al [86] | Protocol | ED care coordination (ED nurse led) | No bedded capacity. | NA | ED | None | Determined to be vulnerable or frail by an emergency nurse using the Clinical Frailty Scale (CFS) at the time of an ED visit. CFS cut off not provided.   Exclusion  Care home residents | Not reported | NA | ED nurses who receive additional didactic and experiential training from emergency physicians, geriatricians, pallia- tive care physicians, social workers, pharmacists, physical therapists, and occupational therapists. They are certified by the Nurses Improving Care for Health system Elders (NICHE) program | 8 transitional care nurses are scheduled complete assessments and provide care coordination for older patients.   The TCNs assess patients for symptoms of delirium, cognitive impairment, functional limitations, polypharmacy, caregiver strain, and fall risk, followed by referral and ED-based care and coordination during and following patients' ED visits. Patients who are discharged from the ED receive a follow up call from a TCN within 3 days of discharge from the ED | NA | NA |
| Ngian et al [105] | Observational cohort (no comparator) | AFU within ED CDU | 14 beds | Not reported | ED | Not reported | Reference to written referral criteria. Unclear how these were operationalised. | Not reported. Operational between 10 a.m. to 6 p.m. during weekdays and 10 a.m. to 4 p.m. during weekends. | Mean age; 83  Diagnosis not reported | Geriatrician led. Consultant remotely supervising a Geriatric Medicine trainee based solely in ED. Physiotherapist, OT, SW. . | "rapid" geriatric medical assessment model based in emergency department. | Not reported | Not reported |
| O'Shaughnessy et al [106] | Observational cohort (no comparator) | ED care coordination ( nurse led) | No bedded capacity. | NA | ED | 70 | Manchester Triage Score (MTS) of urgent, standard or non-urgent. | Screened by frailty team. Patients could be assessed by specialist nurse prior to ED assessment. Operational Monday to Friday (8:00 – 18:00), | Mean age; 80  Musculoskeletal 24% Respiratory 11% Circulatory 11% Infection 9% | Advanced nurse practitioner, physiotherapist, OT, SW, all with specialist training and competencies in the care of the older person. Geriatrician provides advice and clinical review as required | Clinical ownership with ED team who take their recommendations into account in order to establish the disposition. When the ED disposition is discharge, team recommend the most appropriate out-of-hospital pathway and facilitate referrals to and communication with those services. | Not reported | Not reported |
| Pajera et al [107] | Observational cohort (no comparator) | AFU within ED CDU | 6 beds | No | ED |  | Expected LOS 48-72 hours Exacerbation of chronic illness Functional impairment necessitating CGA | Not reported | Mean age; 86  CHF 27%, Respiratory infection 37%, Confusional syndrome 14% | Geriatrician-led. Further details not provided | Functional assessment was performed using the Red Cross physical disability (CRF) and mental disability (CRM) scales and the Barthel index. | Not reported | Not reported |
| Puig et al [96] | Before and after (uncontrolled) | AFU within ED CDU | 13 beds | Yes   "Environmental; adapted area for patients identified as frail " | ED | Not reported | Frail patients requiring observation with no intensive supervision. Unclear how these clinical criteria were operationalised. | Electronic alert generated in ED triage using a population based health markers. It generates a triage alert for each patient who arrives in the ED. Supplemented by clinical assessment at triage for diseases associated with frailty | Not reported | ED team with specialist training. Further details not provided. | Identified patients receive CGA by members of the ED team that have received additional training. | Not reported | Not reported |
| Roussel-Laudrin et al [108] | Observational cohort (no comparator) | Geriatrician in ED | No bedded capacity | NA | ED | 75 | At least two acute medical pathologies. | Not reported | Mean age; 86  Falls 30% Neuropsychiatric 17.4% General decline 12.6% | Geriatrician. | Assessment by geriatrician in ED using a pre-established grid. Physical function assessed using six point ADL score and gait assessed using timed up and go test. Cognitive function assessed using SPMSQ. | Not reported | Not reported |
| Sophia et al [97] | Before and after (uncontrolled) | Geriatrician in ED | No bedded capacity | NA | ED | 80 | No written eligibility criteria  Excluded   ACS Stroke Severe sepsis Fracture | Not reported   Operational Monday to Friday core working hours (09:00 – 1700), | Mean age not reported. | Geriaction led. Physiotherapist. OT | Not reported | Not reported | Not reported |
| Tan et al [110] | Observational cohort (no comparator) | Geriatrician in ED | No bedded capacity. | NA | ED | Not reported | Not reported | Referred at discretion of "senior" ED physician if felt to benefit from CGA. including patients with multiple medical diagnoses, frailty, dementia, delirium, falls, syncope and other common presentations in older adults  Operational Monday to Friday core working hours (09:00 – 1700), | Mean age; 85  Diagnosis not reported | Geriatrician led. Three consultant-led and two senior trainee-led sessions per week in the ED. Physiotherapist, OT and SW. | Not reported | Not reported | Not reported |
| Southerl et al [109] | Observational cohort (no comparator) | AFU within ED CDU | 8 beds. | No | ED | >65 | Not reported | Admitted to unit when further diagnostic tests or monitoring needed prior to a safe discharge, but the patient does not immediately meet threshold for admission criteria.   Admitted to observation unit at the discretion of the attending ED physician. Referral to geriatrician at the ED discretion. Unclear how these criteria were operationalise in practice.   Geriatrician consult team was available Monday through Saturday | Mean age; 73 | Nurse case managers, Physiotherapist. Pharmacist medication reviews by ED pharmacists were initiated 6 months into the study period and were available on weekdays only. | Not reported | Not reported | Not reported |
| Wallis et al [98]  Marsden et al [95] | Non-randomised controlled trial | ED care coordination (ED nurse led) | No bedded capacity. | NA | ED | 70 | No specific written criteria   Exclusion  None | Referrals received directly from ED staff or via routine electronic record system rounding. Frailty screening of patients was initially trialled but found to be of limited benefit. Exact criteria for assessment not reported.   Operational 7 days a week ( weekdays 0700 to 1730) (weekend 0700 to 1530) | Mean age; 81  Cardiac 26% Trauma 18% Gastrointestinal 9% | Specialist nurses had a minimum of five years post registration experience and specialist expertise and/or education in both emergency nursing and care of the older person | Clinical ownership remains with ED. Specialist nurses operate as a supplementary sub-speciality team assisting the primary ED nurses and physicians.  Provide targeted geriatric assessment using elements of CGA. Help fast-track diagnostic processes and engage the MDT. Facilitate admission to a specialty in-patient ward when appropriate. Can liaise with appropriate community services to support discharge. | Not reported | Not reported |

Table 2D Care Home Models

| **Author** | **Study type** | **Where** | **Source of referral** | **Specific condition** | **Process of referral** | **Eligibility criteria** | **Population** | **Who: Team and training** | **Clinical governance** | **Treatment and diagnostics** | **Telehealth enabled** | **Out of hours arrangements** | **Standard of care (work as imagined)** | **Frequency of assessment and intervention (work as done)** | **Proportion of eligible patients assessed** |
| --- | --- | --- | --- | --- | --- | --- | --- | --- | --- | --- | --- | --- | --- | --- | --- |
| Brickman et al [114] | Time-series | Single care care home with 90 beds. Beds were a mixture of acute and long-term nursing care. | NH | No | Care home staff perform initial acute triage. The criteria which trigger acute assessment are not reported. On site advanced nurse specialists and physician associates resident on site then provide acute assessment | Not reported. | Not reported | Acute care delivered on site by specialist nurse or physician associate. | Not reported. In the event the patient is unstable and/or requires transfer to hospital, the on-call ED physician is notified and decision on disposition is jointly determined. | Access to IV fluids, medications. States access to imaging studies and laboratory tests but how these obtained not described. | No | Advanced practioner on site 24 hours with access access to an ED physician by phone. In the event the patient requires transfer to hospital the on-call emergency physician is notified and decision on disposition (transfer to ED or alternate plan) is determined. | Advanced practioner provide basic management utilizing problem-based protocols. These protocols use "universally accepted standard-of-care for acute presentations (chest pain, altered mental status, shortness of breath, abdominal pain, fever, syncope, hypoglycaemia, minor trauma) | Frequency of assessment and number of patients receiving individual interventions not reported. | The frequency of medical review and number of patients assessed is not reported |
| Crilly et al [115, 116] | Obseravtional cohort | Service operated from within a signle hospital and was offered to 42 low- and high-care NH within the region. | ED | No | Hospital out-reach service. Accepted referrals five days a week (0800-1600). After hours and on weekends, if the patient was suitable for inclusion, they stayed in the ED short stay unit and were reviewed by the HINH nurse on the ensuing weekday. | Illness that required hospital services but not necessarily in-hospital admission. This was not further defined | Mean age; 86  Respiratory (24%) Cellulitis (18%) Urinary tract (18%) | Consisted of emergency nurses trained to facilitate care transitions of older adults in the ED to the community. | Required signed request from usual general practioner prior to team review. Unclear who has medical responsibility for patient during the episode of care | Could include delivery of intravenous fluids and intravenous antibiotics. | No | Not reported | Specialist delivers acute care nursing support services, medication and equipment to the ACF registered nurse and/or enrolled nurse. These services may include initial training and education regarding antibiotic or intravenous fluid administration. Although not explicitly stated treatment appears to be delivered by NH team with support from HaH team.   The HaH nurse checks with the care home registered nurse and patient on the patients’ progress initially on a daily basis and then every couple of days. | Frequency of assessment and number of patients receiving individual interventions not reported. | 62 residents were enrolled in the HINH programme during the first 12 months that the programme was operational. The proportion of patients admitted from care homes in which the service was operational is not reported. |
| Joseph et al [118] | Observational cohort | 6 care homes within the catchment of a single hospital. | NH | No | The service is used for acute evaluations when facility staff judged that patients would otherwise require ED transfer. The criteria under which care home nurse would contact ED physician are not reported | Not reported. | Mean age; 75 | Clinical care specialist (CCS) who is a paramedic or emergency medical technician. Not entirely clear whether a CSS was placed at each site or a single CSS covered all 6 sites. | Not reported | The CCS uses a cart with point- of-care labs, electrocardiograms, telemetry, and ultrasound.  Patients can also be directly transported for outpatient imaging (eg, chest radiograph and computed tomography. | Yes  Stated on demand telemedicine consultation by ED physician. The nature of the telemedicine intervention not reported. | CSS available on site at NH 24 hours a day. Physician support available 24 hours via phone. | Order sets and pathways are used to streamline decisions to treat in place or transfer. The order sets are not described. | Frequency of assessment and number of patients receiving individual interventions not reported. | 2311 patients were evaluated by the service over a 12 months period. Care was escalated to hospital in around a quarter of all patients. Most common presentations were hear failure, COPD and diabetes. A third of patients with DM as main compliant were escalated. |
| Lau et al [117] | Observational cohort | 38 care homes could access the service | ED  GP |  | Process of referral unclear. Referrals made to a dedicated phone line. Criteria triggering referral from care home unclear.   Patients could be assessed in the ED. ED Short Stay Unit adjacent to the emergency department for assessment. | Based on clinical judgement of specialist team | Mean age; 83  CAP ( 31.6%) COPD (8.4%) UTI (5.3%) Urinary retention (12.6%) Dehydration (5.3%)  Advanced-stage dementia (4.2%). | Geriatrician led, registrar and nurses. Support from palliative care team. Physiotherapy, SALT, OT | Hospital physician | Ability to provide intravenous fluid / antibiotics / oxygen / anticoagulation. | No | Not reported | The team offered a maximum of daily visits. It is not clear who undertook the visits. | Frequency of assessment and number of patients receiving individual interventions not reported. | Of the 187 referrals to the intervention, after an initial geriatrician assessment, a total of 95 patients were recruited and consented to TRC. Reasons for non- recruitment to TRC included: 57 patients (57/83, 68.7%) who required management as hospital inpatients; 18 patients (18/83, 21.7%) only required management in the Emergency Department; and eight patients (8/83, 9.6%) remained at the facility with GP management |
