# Supplementary material for: Acute care models for older people living with frailty: a systematic review and taxonomy
Source: BMC Geriatr. 2023 Dec 5;23:809. doi: 10.1186/s12877-023-04373-4 (PMC10699071; doi:10.1186/s12877-023-04373-4)
Supplement: Supplementary file 3 — Additional file 3. [file 12877_2023_4373_MOESM3_ESM.docx]

Supplemental table 3

The tables provide a description of the outcomes described in each study and the reported results. Bedded Acute Frailty Units, Hospital at Home models, ED in-reach models and care home models are reported separately

Table 3A Outcome measures and results

Bedded Acute Frailty Unit

| **Author** | **Design** | **Participants** | **Intervention** | **Comparator** | **Primary**  **Outcome** | **Time horizon** | **COMET**  **core area** | **COMET domain** | **Result** | **Secondary outcomes** | **Time horizon** | **COMET core area** | **COMET: domain** | **Result** |
| --- | --- | --- | --- | --- | --- | --- | --- | --- | --- | --- | --- | --- | --- | --- |
| Abdalla et al [24] | Retrospective cohort  Single site Community teaching hospital.  01.07.2013 - 31.08.2013  Used electronic health record to compare incidence of falls in patients admitted to AFU vs usual care. Statistical comparison using zero inflated Poisson regression model | Aged > 65 to AFU unit or general medical unit during study period | n = 941 | n = 6128 | Falls  Falls recorded using the hospital online adverse reporting system | Duration of admission | Adverse event | Fall | Unadjusted Incidence rate ratio 0.18 (0.09-0.37),   Adjusted 0.23 (0.13-0.54) (age / sex / medications / admission falls risk) | NA | NA | NA | NA | NA |
| Abisheganaden et al [25] | Retrospective cohort Multi site Three acute hospitals  01.01.2007 - 31.12.2007  Used clinical and administrative Statistical comparison using logistic regression | Aged > 65 years and older admitted to acute geriatric units and other medical units for pneumonia.   Pneumonia defined usinf ICD-9-CM code with additioanl clinical validation | n = 479 | n = 2242 | All cause mortality | 30 days | Death | Mortality | Adjusted OR. 0.72 (0.52-1.00) | LOS   Subgroup analysis of 30 day mortality > 80 and premorbid functional impairment | 30 days | Resource use | Hospital | LOS   >80 Adjusted OR 0.73 (0.54-0.99) Functional impaired - Adjusted OR 0.65 (0.46-0.93) |
| Ahlund et al [26] | Non-randomised controlled trial Multi-site Two acute hospitals  01.03.2013 - 01.07.2015 | Aged > 75. with frailty as assessed by the FRESH (frailty screening instrument).   Allocation based on availability.  If met eligibility allocated to the control group if no bed available on ACE unit | n = 408 | n = 206 | Function   Function assessed using hydraulic hand dynamometer, functional mobility using the timed up and go test, submaximal aerobic capacity.   Test performed before discharge and at 3 months follow up. The day of initial tesing was not standardised | 3 months | Life impact | Physical functioning | HS Adjusted OR 4.4 (2.2-9.1),  6-MWT (OR 13.9 4.2-46.2) TUG (1.1-5.4) | NA | NA | NA | NA | NA |
| Asplund et al [15] | Randomised controlled trial Single site Acute hospital 18.03.1996 - 08.12.1996 | Aged > 65 years requiring acute medical admission. No specific targeting of frailty.   Randomization temporarily sus- pended when one or both units at capacity. | n = 190 | n = 223  Usual care on. GM ward. Lead by generalist with reduced access to therapists. | Composite outcome (death/severe dependence/psychological well being)  Function assessed using Barthel index at an out-patient hospital visit. Fifteen patients who were unable to attend were interviewed by telephone | 3 months | Death  Life impact | Mortality  Physical functioning | No difference in 3 month outcome or functional outcomes | LOS | Admission to discharge | Resource use | Hospital | No difference |
| Covinsky et al [16] | Randomised controlled trial Single site Acute hospital 01.11.1990 - 01.03.1992 | All patients over 70 with an acute medical illness | n = 327 | n = 324  Usual care consisted of services provided by physicians and nurses in other acute care medical units. The staff of the intervention unit was not involved in the care of patients receiving usual care, and none of the four elements of the program were implemented in usual- care units. Attending physicians and resident physicians provided care to patients in both the intervention and usual-care groups. | Cost | Duration of admission | Resource use | Economic | Duration of admission and service set up costs | NA | NA | **NA** | NA | NA |
| Chittok et al [27] | Retrospective cohort Single site Acute hospital 01.04.2004 - 31.03.2005  Used electronic health record. Statistcal analysis using a cox proportional hazards model to estimate the hazard ratio for prolonged stay | Aged > 75. Consecutive medical admission. Patients transferred between wards excluded. Patients discharged straight from ED excluded | n = 324 | n = 634 | Average LOS | Duration of admission | Resource use | Hospital | ALOS 7.6 in AFU vs 12.4 days in usual care  Increased risk of a prolonged stay in usual care group after adjustment for confounders (adjusted HR 1.10–1.67) | All-cause mortality   Readmission   Discharge destination | 30 days | Death  Resource | Hospital resource | increased discharge to usual place of residence in AFU group. adjusted OR 1.75 ( 1.04–2.96) |
| Ekerstad et al [28] | Non-randomised controlled trial Multi-site Two acute hospitals  01.03.2013 - 01.07.2015 | Aged > 75. with frailty as assessed by the FRESH (frailty screening instrument).   Allocation based on availability.  If met eligbility allocated to the control group if no bed available on ACE unit | n = 206 | n =202 | Functional outcomes using Katz index  A one-step decline on the ADL Staircase, ie loss of independence in one or more ADLs considered clinically relevant. | 3 months | Life imact | Physical functioning | lower risk of decline in ADLs [odds ratio (OR) 0.093; 95% confidence interval (CI) 0.052–0.164; P,0.0001], and with a less prevalent increase in the degree of frailty (OR 0.229; 95% CI 0.131–0.400; P,0.0001) | NA | NA | NA | NA | NA |
| Ekerstad et al [29] | Non-randomised controlled trial Multi-site Two acute hospitals  01.03.2013 - 01.07.2015 | Aged > 75. with frailty as assessed by the FRESH (frailty screening instrument).   Allocation based on availability.  If met eligbility allocated to the control group if no bed available on ACE unit | n = 179 | n =166 | health-related quality of life (HRQoL) using the using the HUI-3 instrument | 3 months | Global quality of life | Global quality of life | Adjustment anaylsysis intervention group were less likely to present with decline in HUI scores in mutliple domains | All-cause mortality  Readmission | 3 months | Death  Resource | Mortality  Resource | Lower 3-month mortality adjusted by Cox regression analysis (hazard ratio [HR] =0.55, 95% CI =0.32–0.96), |
| Ekerstad et al [31] | Non-randomised controlled trial Multi-site Two acute hospitals  01.03.2013 - 01.07.2015 | Aged > 75. with frailty as assessed by the FRESH (frailty screening instrument).   Allocation based on availability.  If met eligibility allocated to the control group if no bed available on ACE unit | n = 67 | n = 72 | Satisfaction  Survey details not provided. | Life imact | Life imact | Patient preference | Significantly more patients in the intervention group responded positively | NA | NA | NA | NA | NA |
| Ekerstad et al [30] | Non-randomised controlled trial Multi-site Two acute hospitals  01.03.2013 - 01.07.2015  Cost-effectiveness analysis, adjusted for confounders using regression.   Data on costs calculate using hopsital administrative database. Health related quality of life mortality index (HRQoL). | Aged > 75. with frailty as assessed by the FRESH (frailty screening instrument).   Allocation based on availability.  If met eligbility allocated to the control group if no bed available on ACE unit | n = 206 | n =202 | adjusted incremental cost-effectiveness ratio | 3 months | Resource use | Economic | The incremental cost difference between the groups, was 3226 US dollars (95% CI: 6167 to 285). | Na | Na |  |  | Na |
| Flood et al [32] | Retrospective cohort  Single-site Two acute hospitals  01.01.2010 - 31.12.2010  Used electronic health record and routinlely collected administarive data. Analysis restricted to the 25 most common diagnoses shared between AFU and usual care groups   Generalized linear regression was used to estimate cost ratios and 95% confidence intervals adjusted for age, sex, comorbidity score, and case mix index (CMI). | Older patients who spent the entirety of their hospital stay in either the ACE or UC unit in fiscal year 2010. Patients were assigned to a unit primarily on bed availability. | n = 428 | n =- 390  Usual care did not include ACE care coordinator or access to volunteers to assist at meal times or with cognitive stimualtion. The same group of hospitalists provided care on the ACE and usual care wards. Hospitalists used the geriatric consultation service for UC patients. | Cost | Duration of admission | Resource use | Economic | Adjusted cost ratios showed cost savings for patients with low (0.82; 95% CI, 0.72-0.94) or moderate (0.74; 95% CI, 0.62- 0.89) CMI scores; care was cost neutral for patients with high CMI scores (1.13; 95% CI, 0.93-1.37). | Readmissions | 30 days | Resource | Hospital | 30 day readmission lower in the AFU group. (7.9% vs 12.8%; P=.02) |
| Goldberg et al [17] | Randomised controlled trial Single-site 01.07.2010-01.12.2011  Impact on specialist unit, with five enhanced components on outcomes in patients with acute medical illness requiring admission complicated by dementia or delirium. 1:1 allocation dependent on bed availability.   A researcher collected information through interviews. Two trained researchers observed the care of 90 randomly subsampled participants. Observations were made every five minutes for six hours per patient. Clinical staff were not aware of which patient was being observed | Aged over 65, identified by physicians in the admissions unit as being “confused” on the acute medical unit. | n = 310 | n = 290 | Number of days at home  Composite outcome (death; time spent in hospital, re-admissions, inpatient rehabilitation or intermediate care; or new placement in a care home) | 90 days | Composite outcome across multiple core areas (Death/Life impact/Recourse use) | Mortality | No significant difference | Quality of Life  Function   Cognitive impairment   Patient and carer satisfaction | Admission to discharge | Life impact   Delivery of care | Global quality of life   Physical functioning   Cognitive function  Patient/carer satisfaction | Patients randomised to the specialist unit had a significantly higher quality of hospital experience. |
| Harris et al [18] | Randomised controlled trial Single site Acute hospital 01.02.1985-01.10.1986  Unblinded. On the day of discharge a multidimensional questionnaire was administered by trained research assistants. | Aged >70. Admitted with acute medical problem from ED. No clinical criteria applied. | n = 97 | n = 170 | Mortality  LOS  Discharge rates to institutional care | 3 monthly intervals for 12 months | Death  Resource use   Life impact | Mortality  Hospital  Physical function | No significant difference | NA | NA | NA | NA | NA |
| Hung et al [33] | Prospective cohort Single centre Acute hospital 01.11.2008 - 01.08.2011  Consultative specilaist acute geriatric care. Interviews on admission to the hospital and post discharge. Combined with medical record review by a clinician investigator who was not blinded to treatment assignment | Aged > 75. Admitted for acute medical care. Those in the intrevention group were receiving care at the outpatient geriatric practice which delievrs primary and geriatric care to older adults. | n = 173 | n = 173 | Readmission | 30 days | Resource use | Hospital | No difference in readmission rate. OR 0.91 (0.39-2.10) | Health care utilisation  Composite hospitalizations, observation unit stays, and emergency room visits  Function  FIM-MOTOR subscale assessed by assessment and interview  Satisfaction 3-item Care Transition Measure (CTM).  Adverse event  Catheter-associated urinary tract infection (UTI), falls, and restraint use. Incidence of adverse events during incident hospitalization was collected through medical record review | 30 days    30 days     30 days    Duration of admission | Resource use  Life impact  Delivery of care  Adverse events | Hospital   Physical function  Patient/carer satisfaction  Falls / pressure ulcers / catheter use | Shorter LOS (0.8 days, ( 0.7-0.9; p=0.001) when compared with patients receiving usual care.   No difference in functional status between the two groups  No difference in satisfaction  Lower odds adverse event in AFU group (9.5% vs. 17.0%; Adjusted OR: 0.11 (0.01-0.88 p=0.04) |
| Jayadevappa et al [34] | Retrospective cohort Single centre Acute hospital 01.01.1999 - 31.12.2002  Data obtained from electronic health record. Cost data obtained from administrative data | Aged > 65. Used diagnostics codes to identify a sub-group a primary diagnosis of CHF, CAP or UTI.   Used a random sample of eligble patients matched to a sample in receipt care on a general medical ward | n = 680 | n = 680  Patients admitted to a general medical ward with target conditions. | Cost   Calculated from hospital charges usings a cost-to-charge ratio of 0.80. | Duration of admission | Resource use | Economic | Mean cost of AFU unit was 9.7% lower than that of non-ACE unit ($13,586 vs. $15,040, P = 0.012). | LOS  Care home admission  Readmissions | Duration of admission   1 year | Resource use | Hospital  Societal | Mean LOS was 1 day shorter for ACE unit (4.9 vs. 5.9 P = 0.01).  Annual readmissions 11% lower in adjusted analysis  No difference in discharges to care homes |
| Landerfield et al [19] | Randomised controlled trial Single site Acute hospital 01.11.1990 - 01.03.1992  Multivariable ordinal logistic-regression model controlling for age, sex, race, the number of basic and instrumental activities of daily living. | All patients over 70 with an acute medical illness. 1143 eligible patients were not enrolled in the study because beds were not available in both the intervention and usual-care units | n = 327 | n = 324  Usual care consisted of services provided by physicians and nurses in other acute care medical units. The staff of the intervention unit was not involved in the care of patients receiving usual care, and none of the four elements of the program were implemented in usual- care units. Attending physicians and resident physicians provided care to patients in both the intervention and usual-care groups. | Function   Home visit. at the hospital or in the patient’s home. Assessed by the ADL Staircase 3 months after discharge from hospital.   This instrument comprises five personal ADL (PADL) items (ie, feeding, transferring, going to the toilet, dressing, and bathing),32 extended by four instrumental ADL (IADL) items. | 3 months | Life impact | Physical functioning | The mean number of ADLs that could be performed independently at hospital discharge were 3.6 for AFU and 3.3 for usual-care group (P value 0.05).   No difference in function at 3 months.   No difference in care home admission at 3 months. | Cost  Total cost estimated administrative data |  | Resource use | Economic | No difference |
| Naughton et al [20] | Randomised controlled trial Single site Acute hospital 01.04.1991-31.12.1991  Unblinded. | Randomised in the ED after decision to admit had been made. Intervention arm admitted under the care of a geriatrician. Control arm admitted under general internist on call. | n = 51 | n = 60 | Cost   Direct costs attributed to laboratory services, occupational therapy and physical therapy, diagnostic services and pharmacy services | Duration of admission | Resource use | Economic | No significant difference | LOS | Duration of admission | Resource use | Hospital | No significant difference |
| Salinas [35] | Retrospective cohort Single site Acute hospital  01.06.2001 - 15.03.2002  Used clinical record review and structured interviews with the patient, caregiver and/or family.   Satistical analysis of binary outcomes with logistic regression. | All patients > 65 admitted with acute medica illness. | n = 68 | n = 75  Allocated to control if team declined intervention or could not provide due to capacity issues.  The usual care group were attended by an internist physician, a medical resident of this specialty, general care nurses, and had access to physical and occupational therapy, and a social worker by means of referral. | Functional  Number of basic activities of daily living (BADL) evaluated using the Katz index | Discharge | Life impact | Physical functioning | Less functional impairment in the AFU group, 19.1%, had functional deterioration on discharge relative to 40% in the usual care group (p = 0.013). | NA | NA | NA | NA | NA |
| Saltvedt et al [21] | Randomised controlled trial Single site Acute hospital  31.10. 1994 -13.11 1995  Secondary analysis of RCT assessing impact on function, symptoms of depression and general well-being. | Patients > 75. Recruited from acute medical ward. At least one target condition from a list of clinical and functional criteria | n = 127 | n = 127 | Function  Barthel Index was used to assess ADL and Lawton Index for IADL | 1 year | Life impact | Physical function | No significant difference | Cognitive function  Assessed using MMSE | 1 year | Life impact | Cognitive function | No significant difference |
| Saltvedt et al [22] | Randomised controlled trial Single site Acute hospital   31.10. 1994 -13.11 1995 | Patients > 75. Recruited from acute medical ward. At least one target condition from a list of clinical and functional criteria | n = 127 | n = 127 | Mortality | 1 year | Death | Mortality | HR 0.39 (95% 0.21–0.72) for marltiy at 3 months. At 12 months. Intervention 28% vs 34% in usual care (p 0.06). A-priori alpha 0.05. | NA | NA | NA | NA | NA |
| Schubert et al [36] | Retrospective cohort Single site Acute hospital 01.01.2012 - 30.09.2014  Used electronic health record. | All patients > 65 admitted with acute medical illness. | n = 421 | n = 372  Patients eligible for intervention positive review but not assessed as the admitting team declined the offer of consultation or no capacity to provide review | LOS   Readmissions  All cause mortality | 30 days | Resource use | Hospital | No significant difference | Cost  Total cost estimated from several sources of administrative data | Admission | Resource use | Economic | No significant difference |
| Stewart et al [37] | Prospective cohort Single  Community teaching hospital Study date not reported  Outcome assessment was based on data collected by trained interviewers who were not involved in patient care. Unblinded. | All patients older than 85 years who required acute care and patients younger than 75 meeting specific criteria including markers of frailty | n = 34 | n =- 27  Attempted to randomise but could not due to bed pressures | LOS   Change in the number of medications. | Duration of admission | Resource use  Delivery of care | Hospital   appropriateness of intervention | No difference in LOS  AFU associated with significant reduction in the number of medications administered | NA | NA | NA | NA | NA |
| Wald et al [38] | Non-randomised controlled trial Acute hospital 02.11.2007-15.04.2008  Recognition of abnormal functional status was determined from chart review and consisted of both the physician’s detection of abnormal functional status and evidence of a corresponding treatment plan  Falls were determined from mandatory event reporting. A two-step, retrospective chart abstraction was employed. |  | n = 122 | n = 95  Usual care included daily discharge planning rounds with a discharge planner and social worker focused exclusively on discharge planning. | Clinical processes  Recognition of abnormal functional status by the primary team. Increased recognition of abnormal functional status; Recognition of abnormal cognitive status and delirium;  Adverse events   Falls Delirium Restrain use | Duration of admission | Delivery of care   Adverse events | Quality and adequacy of intervention. | Patients on the AFU had recognition and treatment of abnormal functional status (68.9% vs 35.8%, P < 0.0001). | LOS  Readmission | 30 days | Resource use | Hospital | No difference |

Table 3B Outcome measures and results

Hospital at Home care models

| **Author** | **Design** | **Participants** | **Intervention** | **Comparator** | **Primary Outcome** | **Time horizon** | **Result** | **COMET: core area** | **COMET: domain** | **Secondary outcomes** | **Time horizon** | **COMET: core area** | **COMET: domain** | **Result** |
| --- | --- | --- | --- | --- | --- | --- | --- | --- | --- | --- | --- | --- | --- | --- |
| Aimonino et al [47] | Randomised controlled trial Single-site 01.04.2004-01.04.2005  Intention to treat analysis. KM models to compare cumulative survival at 6 months follow up. | Aged 75 and older with a diagnosis of acute exacerbation of COPD. | n = 52 | n = 52 | All-cause mortality  Readmission  Cost | 6 months | No difference in mortality.   HaH longer mean LOS (15.5 (9.5) vs 11.0 (7.9) days, P .01)  HaH experienced a lower rate of readmission (42% vs 87%, P <.001), and the time to readmission was longer for hospital-at-home patients (78 vs 37 days)  The mean total cost was $1,175.9 for each patient treated at home and $1,390.9 for patients treated in the hospital (P 5.38). | Death  Resource use | Mortality  Hospital  Economic | Depression status  Function   Cognitive function   Nutrition  Quality of life   Nutrition    Satisfaction and care stress | 6 months | Life domain  Delivery of care | Physical function  Cognitive function  Patient/carer satisfaction | No significant difference |
| Augustine et al [66] | Prospective observational cohort Multi-site 18.11.2014 - 31.08.2017   Assessment of a HaH care model bundled with a 30-day post acute period of home-based transitional care.  Secondary analysis to estimate the confounding and moderating effect of social support on outcome on LOS and 30 day readmission | Aged > 18. Eligible admission diagnoses were acute exacerbations of asthma or chronic obstructive pulmonary disease, decompensated congestive heart failure, urinary tract infection,, community-acquired pneumonia, cellulitis of the lower extremities, deep venous thrombosis or pulmonary embolism, hypertensive urgency, hyperglycaemia, and dehydration. | n = 295 | n = 212 | Impact of social support on LOS and readmission | 30 days | Instrumental social support attenuated the effects of HaH upon any ED visit (base model: OR 0.61, p=0.037; controlling for social support: OR 0.71, p=0.15). | Resource use | Hospital | NA | NA | NA | NA | NA |
| Burton et al [67] | Randomised controlled trial Single site 01.11.2000-30.09.2001  Nested study of patient and family satisfaction within prospective non-randomised trial. | Aged 65 and older residing within a specific catchment area. Care residents excluded. | n = 84 | n = 130 | Satisfaction  40-question survey measuring nine domains of care for patients and a 37-question survey measuring eight domains of care for family members. | 2 weeks after discharge | Patients were more satisfied with treatment in Hospital at Home than with treatment in the acute care hospital in five domains | Care delivery | Patient satisfaction | NA | NA | NA | NA | NA |
| Cai et al [68] | Retrospective cohort study.  Single site 01.10.2011 - 31.12.2015  Used electronic health record to match to usual care patients using a propensity score approach (1:1) | Patients treated in a HaH servive comapred with usual IP care. Congestive heart failure, chronic obstructive pulmonary disease, community-acquired pneumonia, diabetic foot ulcer, and complicated wound care. | n = 127 | n = 694 | Mortality  Readmission   NH admission | 30 days | No difference, | Death  Resource use/ Social function | NH admission | Total cost of the index event | average per person costs were $7,792 for HIH services and $10,960 for traditional inpatient care (P<0.001). | Resource | Economic | Cohort |
| Caplan et al [49] | Randomised controlled trial Single site 01.10.1995-01.02.1997  Comparison of rates of adverse events in patients treated in HaH model. Complications assessed by clinical note review. | Primary target older patients but accepted patients <65. (69% of patients over 65). Majority of patients had infections requiring intravenous antibiotic. | n = 51 | n = 49 | Adverse events  Confusion, falls, urinary incontinence or retention, faecal incontinence or constipation, phlebitis and pressure areas   Satisfaction  Patient carer | 28 days after discharge | Lower incidence of adverse events. confusion (0 v. 20.4% [95% CI, 9.1%--31.7%); P= 0.0005) | Adverse events  Care delivery | Confusion   Urinary incontinence  Pressure ulcers  Patient / carer satisfaction | Mortality | 28 days after discharge | Death | Mortality | No difference |
| Caplan et al [50] | Randomised controlled trial Single site 01.10.1995-01.02.1997  Secondary analysis of previously reported trial with focus on physical and cognitive function. | Primary target older patients but accepted patients <65. (69% of patients over 65). Accepted only if they had been assessed as requiring admission by the relevant medical or surgical team | n = 51 | n = 49 | Functional    Barthel index modified Instrumental Activities of Daily Living (IADL) index" | Assessed on admission and again at discharge | mean instrumental Activities of Daily Living score from admission to discharge (mean + 0.65 vs -0.08)) No statistical difference in other measures of function | Life impact | Physical function | NA | NA | NA | NA | NA |
| Clarke et al [69] | Non-randomised controlled trial Multi-site 01.11.2000-01.09.2002  Secondary analysis of NRCT evaluating cost. Used administarve costs data. All 3 sites used different costing methods. | Aged > 65. Requiring acute hospital admission for community-acquired pneumonia, chronic heart failure, chronic obstructive airways disease, or cellulitis. | n = 165 | n = 286 | Cost | Discharge | Hospital at Home intervention were significantly lower than those of usual acute hospital care (mean [SD], $5081 [$4427] vs $7480 [$8113]; P <.001).   Laboratory and procedure expenditures were lower across all study sites and at each site individually | Resource use | Hospital | NA | NA | NA | NA | NA |
| Dismore et al [51] | Qualitative study 01.06.2014 - 01.01.2016  Secondary analysis of previous program focusing on reasons for acceptance or refusal to received a hospital at home intervention | Patients aged > 35 with COPD acutely admitted with pneumonia and a low risk DECAF score (0–1). COPD. Not focused on frailty although average age was > 65. | 89 survey responses. 44 patients, 15 carers, 15 physicians, 11 specialist nurses and 4 managers | NA | Qualitative | Discharge | A common reason for declining participation in the RCT was fear of being alone when unwell, even when offered (same day) social support. | Care delivery | Patient satisfaction | NA | NA | NA | NA | NA |
| Federman et al [70] | Prospective observational cohort Multi-site 18.11.2014 - 31.08.2017   Assessment of a HaH care model bundled with a 30-day post acute period of home-based transitional care.  Used electronic health record supplemented by chart review.  Matched using inverse probability weighting (IPW) | Aged > 18. Eligible admission diagnoses were acute exacerbations of asthma or chronic obstructive pulmonary disease, decompensated congestive heart failure, urinary tract infection,, community-acquired pneumonia, cellulitis of the lower extremities, deep venous thrombosis or pulmonary embolism, hypertensive urgency, hyperglycaemia, and dehydration. | n = 295 | n = 212 | LOS   All-cause hospital readmissions or stand- alone ED visits)   NH admissions  Referral for POC | 30 days | LOS was shorter for HaH patients than con trols (3.2 days vs 5.5 days; difference, −2.3 days; 95% CI, −1.8 to −2.7 days; P < .001). In weighted and adjusted regression analyses, the differences in LOS between HaH and control patients was −2.41 (0.11) (P < .001).  Lower 30 day mortality (8.6% [25] vs 15.6% [32]; difference, −7.0%; 95% CI, −12.9% to −1.1%; P = .01) | Resource use | Hospital | Satisfaction | Ratings of care using HCAHPS survey | Care delivery | Patient satisfaction | Improved satisfaction. More likely to provide the highest rating for overall hospital care (68.8% [119] vs 45.3% [67]; difference, 23.5%; 95% CI, 12.9%-34.1%; P < .001) |
| Gonzalez Barcala et al [71] | Prospective cohort 08.12.2003- 01.01.2010. | matched patients admitted to the hospital during the same period who met the clinical criteria for HAH but who were assigned to CHC because they failed to meet the residential, social or willingness criteria. | n = 25 | n = 50 | Mortality  LOS  Readmission | 30 day and 1 year | HAH patients shorter LOS mean of (7.0 days vs 12.2 days) | Death  Resource use | Hospital | Satisfaction | Discharge | Care delivery | Patient satisfaction | Unable to asses |
| Greenough et al [72] | Non-randomised controlled trial Multi-site 01.11.2000-01.09.2002  Survey questionnaire completed as a component of a prospective, nonrandomized clinical trial of a HaH care model focused on patient and carer satisfaction | Eligible patients were community-dwelling persons 65 years and older residing within a specific catchment area who required acute hospital admission for specific diagnosis | n = 84 baseline interview,  n = 74 interview at 2 weeks | n = 57 baseline interview,  n = 47 interview at 2 weeks | Satisfaction  Fifteen-question survey questionnaire asking family members whether they experienced a potentially stressful situation and, if so, whether stress was as- sociated with the situation while the patient received care | 2 weeks after discharge | The mean and median number of experiences, of a possible 15, that caused stress for family members of HaH patients was significantly lower than for family members of acute care hospital patients (mean 􏰈 standard deviation 1.7 􏰈 1.8 vs 4.3 􏰈 3.1, Po.001; median 1 vs 4, Po.001). HaH care was associated with lower odds | Care delivery | Patient satisfaction | NA | NA | NA | NA | NA |
| Greenough et al [73] | Non-randomised controlled trial Multi-site 01.11.2000-01.09.2002  Survey questionnaire completed as a component of a prospective, nonrandomized clinical trial of a HaH care model focused on function | Eligible patients were community-dwelling persons 65 years and older residing within a specific catchment area who required acute hospital admission for specific diagnosis | n = 84 baseline interview  n = 64 interview at 2 weeks | n = 57 baseline interview,  n = 40 interview at 2 weeks | Function  Survey questionnaire. five activities of daily living (ADLs) seven IADLs | 2 weeks after discharge | Patients treated in HaH experienced modest improvements in performance scores, whereas those treated in the acute care hospital declined (ADL, 0.39 vs 0.60,) | Life impact | Physical function | NA | NAs | NA | NA | NA |
| Leff et al [74] | Prospective cohort study Multi-site 30.09.1996-12.02.1998 | Aged > 65. Requiring acute hospital admission for community-acquired pneumonia, chronic heart failure, chronic obstructive airways disease, or cellulitis. | n = 17 | n = 122 | LOS  Safety | Duration of admission | No difference in LOS. | Resource use | Hospital | Cost | Duration of admission | Resource use | Economic | Charges for patients treated in HH were 53% of acute hospital charges |
| Isaia et al [73] | Prospective cohort study Single site 01.02.2007-01.04.2007 | Aged > 75, consecutively admitted from the ED to HaH or usual care. Must not have delirium at point of admission according to CAM but have an intermediate or high risk of developing delirium during the course of admission. | n = 84 | n = 60 | Mortality   NH placement  Adverse events   Delirium   Psychoactive drug use | 6 months | Lowe incidence of delirium in HaH group ( 4.7% vs 16.6%) relative risk of 3.8 (CI 8– 13.72) | Adverse events | Delirium | NA | NA | NA | NA | NA |
| Leff et al [75] | Non-randomised controlled trial 01.11.2001- 01.09.2002  To assess the clinical feasibility and efficacy of providing acute hospital-level care in a patient’s home in a hospital at home. | Aged > 65. Requiring acute hospital admission for community-acquired pneumonia, chronic heart failure, chronic obstructive airways disease, or cellulitis. | n = 84 | n = 73 | Clinical process measures;  Time to intervention number of interventions.  Incidence delirium: using | Duration of admission | HaH had a shorter length of stay (3.2 vs. 4.9 days) (P 􏰆 0.00 (intention to treat analysis | Resource use  Care delivery  Adverse events | Delirium | Cost | Duration of admission | Resource use | Economic | Total costs lower |
| Levine et al [53] | Randomised controlled trial 12.06.2017-16.01.2018. Multi-site  To compare outcomes of home hospital versus usual hospital care for patients requiring admission.  Randomization was stratified by infection, heart failure, chronic obstructive pulmonary disease or asthma, and other diagnose | Aged > 18. Primary diagnosis of any infection, heart failure exacerbation, chronic obstructive pulmonary disease exacerbation, asthma exacerbation, or selected other conditions requiring acute medical admission | n = 43 | n = 48 | Cost | NA | Adjusted mean cost was 38% lower (95% CI, 24% to 49% lower; P < 0.001). | Resource | Economic | Function  Health care use  Readmission | Functioned defined using physical activity during the acute care episode measured using wearable devices.   Health care utilisation during acute epsidoes involved use of laboratory orders, radiology studies, consultations, and length of stay.   Study staff interviewed patients on admission, at discharge, and 30 days after discharge. | Life Impact  Resource use  Care delivery | Physical function  Hospital  Patient satisfaction | Compared with usual care patients, home patients had fewer laboratory orders (median per admission, 3 vs. 15), imaging studies (median, 14% vs. 44%), and consultations (median, 2% vs. 31%). Home patients spent a smaller proportion of the day sedentary (median, 12% vs. 23%) or lying down (median, 18% vs. 55%) and were readmitted less frequently within 30 days (7% vs. 23%). |
| Levine et al [54] | Randomized controlled trial Single site . | Adults admitted via the emergency department with any infection or exacerbation of heart failure, chronic obstructive pulmonary disease, or asthma | n = 9 | n = 11 | Cost | 30 days post admission | Median direct cost of the acute care episode for home patients was 52% (IQR, 28%; p = 0.05) lower than for control patients. | Resource | Economic | Function | 30 days post admission | Life impact | Physical function | More physically active (median minutes, 209 vs. 78; p < 0.01) |
| Levine et al [55] | Randomised controlled trial 12.06.2017-16.01.2018. Multi-site  Qualitative study of previously reported RCT. Establish perceptions of home hospital and hospital bed based care to better describe the different perceptions of care in both settings. | 112 randomised in original trial. 19 of 35 interviewed in the intervention. Control 17/46 | n = 19 | n = 17 | NA | NA | Compared to control patients, home patients had better experiences with their care team, had more experiences promoting healing such as better sleep and physical activity, and had better experiences with systems factors such as the admission processes. | Care delivery | Satisfaction | NA | NA | NA | NA | NA |
| Mas et al [83] | Prospective cohort study 01.12.2015-30.06.2016 2 sites within same provider | Older patients with chronic conditions attended at the emergency department or day hospital for an acute medical crisis. Allocation based on the availability of resources, on caregiver availability, and on patient acceptance | n = 57 | n = 114 | Composite outcome   "health crisis" (recovery from the acute health crisis, referral to an acute hospital, or death), | 30 days post discharge | No difference | Death |  | LOS  Relative functional gain.   Readmission  Mortality. | 30 days | Resource use  Life impact | Physical function | HaH group longer LOS. 9.7 vs 8.2 p < .01).  No difference in mortality or readmission rate |
| Marsteller et al [77] | Qualitative study 01.11.2001-01.09.2002  Qualitative study. Evaluate the HaH model from the perspective of the physicians and nurses delivering care. Isolate concepts at the patient, provider and study level. Used multivariate general estimating equations regression analyses of a patient-specific survey. | Participants were treating physicians who pro vided HaH care and the nurses who provided the continuous direct nursing supervision for the initial portion of a HaH admission | 11 physicians  26 nurses | Na | problems with care; benefits; problem-free index. | Post discharge | Positive effects included quicker patient functional recovery, greater opportunities for patient teaching, and in creased communication with family caregivers. | Care delivery | Patient satisfaction | NA | NA | NA | NA | NA |
| Mendoza et al [57] | Randomised controlled trial Single site 01.05.2006-01.03.2007  Assess whether 'Hospital at home' care model as an effective alternative in the management of decompensated chronic heart failure | Aged > 65 years with a confirmed diagnosis of HF performed at least 12 months prior to the study, who were in New York Heart Association (NYHA) functional class II or III prior to the current acute exacerbation | n = 37 | n = 34 | Mortality  Readmission | 1 year | No difference.   Death or re-admission due to HF or another cardiovascular event occurred in 19 patients in IHC and 20 in HaH (P 1⁄4 0.88 | Death  Resource | Readmission | Variations in functional status (Barthel index) and health-related quality of life (SF-36), since first admission up to 12 months later were also studied. | 1 year | Life impact | Physical function | No difference |
| Echevarria et al [52] | Randomised controlled trial Multi-site 01.06.2014 - 01.01.2016 | Patients aged > 35 with COPD acutely admitted with pneumonia and a low risk DECAF score (0–1). COPD. Not focused on frailty although average age was > 65. | n = 62 | n = 57 | Cost | 90 days | The mean health and formal social care cost was £1016 lower in HAH than in Usual care group.   Wide variation in costs and the one-sided 95% CI crossed both the no effect limit (0) and the prespecified non-inferiority limit of £150 | Resource use | Economic | Mortality  LOS  Readmission   Health-related quality of life." | 90 days | Death  Resource use  Global quality of life |  | No difference in mortality  Reduced LOS |
| Patel et al [87] | Randomised controlled trial Single site 01.04.2004-01.05.2006  Treatment of decompensated HF at home vs usual hospital care. | No age restriction. Established diagnosis of HF. Patients received up to 48 hours of treatment in the ED or medical ward prior to home treatment | n = 13 | n = 18 | Cost  Direct costs (time costs of specialist nurse visits (vist and transpiration), physician cost) | 12 months | The total cost related to CHF was lower in the HC group (p=0.05). | Resource use | Economic | "Clinical status"  NT-proBNP / weight and medical treatment   Assessed by interview and access to medical records | 1 year | Delivery of care | Quality and adequacy of intervention | No difference |
| Saenger et al [83] | Qualiative study 01.09.2014-31.08.2017  Analysis of reasons for acceptance or refusal to received a hospital at home intervention | Aged > 18. Eligible admission diagnoses were acute exacerbations of asthma or chronic obstructive pulmonary disease, decompensated congestive heart failure, urinary tract infection,, community-acquired pneumonia, cellulitis of the lower extremities, deep venous thrombosis or pulmonary embolism, hypertensive urgency, hyperglycaemia, and dehydration. | n= 442 |  | Reasons for participating or not participating in HaH | NA | Of patients who decided to enrol in HaH, most (78%) said they did so because they anticipated being more comfortable at home, and 41% said they chose HaH because they liked having family around.  The most commonly cited specific reason for refusing HaH was concern about the ability of HaH to meet their care needs (13%) | NA | NA | NA | NA | NA | NA | NA |
| Sheppered et al [59] | Randomised controlled trial Multi-site 01.06.2014 - 01.01.2016  Admission avoidance HaH versus hospital admission with CGA when available using 2:1 randomization. Statitical analysis using a used a log-Poisson generalized linear mixed-effects model | Aged over 65 referred to a geriatrician led HaH led service with ability to perform CGA. | n = 700 | n = 355 | Living at home at 6 months. (inverse of death or long-term residential care) | 6 months | 528 of 672 (78.6%) participants in the HAH group versus 247 of 328 (75.3%) participants in the usual care group were living at home (relative risk [RR], 1.05 [95% CI, 0.95 to 1.15]; P = 0.36) | Composite outcome   Life impact   Resource use  Care delivery | Hospital  LOS | Each component of composite measured separately. Cognitive impairment (measured using the Montreal Cognitive Assessment),   Activities of daily living (measured using the Barthel Index)   Readmission or transfer to hospital (also measured at 1 month), | 6 months | Adverse events  Life impact | Delirium  Physical function  Cognitive function | No difference in the presence of delirium risk of cognitive impairment of measurements of activities of daily living  Increased risk for readmission or transfer to hospital in the CGA HAH    Participants who received CGA HAH reported higher levels of satisfaction |
| Skojt-Arkll et al [60] | Randomised controlled trial 30.06.2015  To evaluate whether the patient’s own GP is more effective than a hospital specialist at reducing hospital admissions without affecting the recovery or death rates in elderly patients with acute medical conditions cared for in a HaH model. | Admission rates in a general practitioner- based versus a hospital specialist based, hospital-at-home model: ACCESS, an open- labelled randomised clinical trial of effectiveness | n = 67 | n = 63 | Mortality  Readmissions | 3 months | More patients were admitted in the hospital specialist arm, 29 (45%) versus 16 (24%) of the patients in the GP arm within the first 7 days (Effect size 2.7, 95% CI 1.3–5.8;) | Death  Resource use | Hospital | Functional | EQ-5D,  DEMMI,  OMC,  Chair-stand test Grip strength | Life impact | Physical function | No difference |
| Tibaldi et al [61] | Randomised controlled trial Single site 01.02.1999- 01.04.2002  Trial to assess the impact of HaH care on behavioural issues in patients with severe dementia | Accepted patients with undifferentiated medical conditions needing hospitalization but not expected to require emergency interventions | n = 56 | n = 53 | Behavioural problems  Caregiver’s stress | Discharge | Reduction in " behavioural disturbances" in HaH group. Lower use of anti-psychotic drugs in HaH group. | Adverse events | Delirium | NA | NA | NA | NA | NA |
| Tibaldi et al [62] | Randomised controlled trial Single site 01.04.2002-01.04.2004  Role of HaH in the management of decompensated CHF in older patients.   . | Aged > 75. CHF and a persistent functional impairment indicative of New York Heart Association (NYHA) class III or IV stat when deemed to require admission from ED, need for intravenous drug infusion | n = 48 | n = 53 | Mortality   Readmissions   Time to first re-admission | 6 months | No difference in mortality or readmission at 6 months, | Death  Resource use |  | Depression,  nutritional status Quality-of-life scores. | 6 months | Life impact  Global quality of life | Cognitive function | Patients experienced improvements in depression, nutritional status, and quality-of-life scores. |
| Tsiachristas et al [78] | Retrospective cohort study Multi-site 01.08.2014- 01.12.2016  Three separate services in Scotland. Used electronic medical record. Propensity score matching in combination with regression analysis. | Aged > 65s were classified as an unscheduled admission to general or geriatric medicine. Three geriatrician-led admission avoidance hospital-at-home services in Scotland. | Matched: Site 1 n = 1969 Site 2 n = 925 Site 3 n = 426 | n - 11571 n = 3849 n =- 1683 | Cost   Cost derived from the Cost-It tool of the WHO | Discharge | The cost of providing hospital-at-home varied between the three sites from £628 to £2928 per admission, and costs were driven primarily by staff costs. | Resource use | Economic | Mortality | Death |  |  | Increases risk in mortality in HaH group. (site 1: relative risk 1.09; 95% CI 1.00 to 1.19) (site 2: relative risk 1.29; 95%CI: 1.15 to 1.44) (site 3: relative risk 1.27; 95%CI: 1.06 to 1.54) |

Table 3C Outcome measures and results

ED in reach models

| **Author** | **Design** | **Participants** | **Intervention** | **Comparator** | **Primary Outcome** | **Time horizon** | **COMET: core area** | **COMET: domain** | **Result** | **Secondary outcomes** | **Time horizon** | **COMET: core area** | **COMET: domain** | **Result** |
| --- | --- | --- | --- | --- | --- | --- | --- | --- | --- | --- | --- | --- | --- | --- |
| Alakare et al [84] | Randomised controlled trial Single site   Randomised to the intervention and the control groups with a 1:1 allocation ratio.  Primary outcome established using electronic health record. Secondary outcomes assessed by phone interview. | Patient attending ED. Age ≥ 75 years with features of frailty. Frailty was defined using CFS level. Score of > 4 used to define frailty | n = 213 | n = 219 | Cumulative LOS  Total number of overnight stays in hospital wards (both at the tertiary hospital and the community hospital ward) during 365-day follow-up from the date of the enrolment. | 1 year | Resource use | Hospital | No statistical difference  Intervention and control groups were: 3470 and 3149 days, rate ratio of 1.10 (95 % confidence interval, 0.55–2.19, P = .78). | Cumulative number of admissions to hospital   Readmissions   HRQoL | Readmissions at 3 days, 30 days and 1 year. | Resource use  Global quality of life | Hospital | No significant differences were observed between the groups for any outcomes. |
| Ardents et al [87] | Before-after (uncontrolled) Single site 01.07.2016 - 01.06.2017  Used EMR to assess outcomes in all patients presenting following a fall to ED during 2 6 months implementation periods. Used hospital level data. Unclear what proportion of patients received the intervention. | Patients presenting to ED following a fall. | n = 562 (period 1)  n = 873 (period 2) | Not reported | Direct discharge from ED | Discharge | Delivery of care | Need for further intervention | There was a significant increase in the proportion of patients discharged across the study. Proportion not reported. | Readmission  Average LOS  Staff perception | 28 days | Resource use    Delivery of care | Hospital  Staff perception | No difference in readmission rare. Statistically significant reduction in ED and total hospital LOS.   Not interpretable |
| Basic et al [85] | Randomised controlled trial Single site 01.01.1996-01.01.1997  ED care coordination by specialist nurse. The baseline assessment was communicated to downstream team and care coordinated discharge in intervention group. This information was withheld in the control group. Unblinded to group-allocation   Logistic regression to determine the probability of admission and cox-proportional hazards to assess LOS | Patients presenting to ED. Older people presenting to the ED who were "not severely ill". No specific age criteria. | n = 114 | n = 110 | Admission to hospital.   % assessed by geriatrician   Average LOS | Admission to discharge | Delivery of care  Resource use | Need for further intervention /  Quality and adequacy of intervention  Hospital | No significant effects on admission to the hospital (OR, 0.7; 95% CI, 0.3– 1.7), LOS (hazard ratio [HR], 1.1; 95% CI, 0.7– 1.5) or f.   Assessment did not influence the rate of referral to geriatricians (in those not admitted to a geriatric ward) | Functional   Defined a priori as a decrease in the total MBI score > 3 | Admission to discharge | Life impact | Physical function | No difference in rates of functional decline |
| Buttery et al [88] | Before and after study Single site 01.08.2014--31.09.2014   Used hospital level data. Historical control. Comparison between 2 months (pre and post intervention). LOS controlled using national LOS benchmarking data. | All medical admisions > 75 screened. | n = 46 (Post) | n = 49 (Pre) | Processes of care   % identified with frailty who had issues identified addressed,   % referred to COTE,   mean LOS prior to transfer to COTE ward | Admission to discharge | Delivery of care  Resource use | Need for further intervention / Quality and adequacy of intervention  Hospital | Improvement in process outcomes and number of addressed problems.   Reduction in average LOS relative to national averages  Mean LOS fell by 4 days | NA | NA | NA | NA | NA |
| Conroy et al [89] | Before and after (uncontrolled) CGA unit within the ED.  2010-2012  Hospital level analysis with data collected on a monthly basis. | The study was limited to reporting outcomes in patients aged >85 years as these were felt to best represent the target population. | n = 461 (Post) | n = 444 (Pre) | Direct discharge from ED | Admission to discharge | Delivery of care | Need for further intervention | The proportion of people aged 85+ admitted in the intervention period was 61.2% (57.7–64.7%) compared with 69.6% (66.0– 73.1%) in the control period, P < 0.001. | LOS  Readmission  Mortality | 7, 30, 90 days  12 months | Resource use  Death | Hospital  Mortality | The mean LOS increased 11.1 days vs 8.9 in control period.  Readmission rates fell across all age groups with risk ratios of 0.71 310 (0.42–1.1) at 7 days, 0.74 (0.55–1.00) at 30 days and 0.77 (0.63–0.93) at 90 days for those aged 85+ |
| Ellis et al [90] | Before and after study (controlled) Single site 01.10.2009-01.02.2010   Designed to be an evaluation of a new service development rather than a test of clinical effectiveness | Aged > 65. Deemed by ED to require admission. The process by which patients were selected to for the observation as opposed to a general medical bed directly is not described. | n = 210 (intervention)  n = 327 (Post control group) | n = 212  (pre-intervention) | Process of care  % access to specialty beds on day of admission,  LOS in a non-specialty bed  acute and total length of stay  Readmission rates (so called ‘‘failed discharges’’)   Clinical outcomes  Mortality  NH admission to residential care   ‘living at home’ (the inverse of death and admission to residential care). | 7 day readmission  30 day readmission  Mortality /NH / living at home at 12 months | Delivery of care  Resource use  Death  Life impact | Need for further intervention  Mortality | Same day discharge rates increased to 17.1% from 1.4% and 7.7% for the ‘‘after’’ control group (P < 0.0005).  Comparison remained significant despite the improvement in the proportion same day discharges (P = 0.001).   Total length of stay was similar for the three groups (12.2 days ‘‘before’’, vs. 12.7 days ‘‘intervention’’, P = 0.78; 11.7 days ‘‘after’’ vs. 106 12.7 days ‘  No statistically significant difference in readmission rates. | NA | NA | NA | NA | Na |
| Foo et al [91] | Before-after study (uncontrolled) Single site 25-12-2006-31.12.2007  Intervention group received geriatric assessment and intervention in ED. Control group received usual care | Aged > 65 years old assessed on the unit and sent home on the same day based on patients with 13 specific diagnoses. Not all listed. | n = 249 | n = 118 | Falls   ED re-attendance and hospitalisation obtained through electronic records. | 3, 6, 9 and 12 months. | Adverse events    Resource use | Falls   Hospital | Reduction in incidence of falls. 41% (adjusted IRR 0.59, 95% confidence interval (CI) 0.48–0.71) and 36% (adjusted IRR 0.64, 95% CI 0.51–0.79) decrease, respectively, at 12 months, having adjusted for age, sex, EDOU diagnosis and baseline BADL. | Function | Telephone interview | Life domain | Physical function | Significant dropout rates in telephone interviews (31.4% control, 21.0% intervention at 12 months). Functional outcomes were therefore not assessed. |
| Kwon et al [89] | Before-after (uncontrolled)  Single site 01.11.2015-01.11.2017  Historically matched control (age / sex / ethnicity). Prior to the intervention the ED team and connection to community resources by the social worker or case manager on duty. | Patients who were admitted to hospital or transferred to a care home were excluded. | n = 283 | n = 283 | Representation and readmission | 30 days | Resource use | Hospital | No difference in rate of ED representation.   Patients in the usual care group that represented to hospital were more likely to be admitted. | Na | Na | Na | Na | Not reported |
| Hwang [92] | Prospective cohort  multi-site 01.01.2013 - 30.07.2015  Data were collected from electronic health record. Controlled for confounding using entropy balancing. Analysis not pooled. | Patients over 65 admitted to the ED. Identification of Senior At Risk score (ISAR)27 scores ≥4, Emergency Severity Index (ESI)28 ≥3, hospital discharge 30 days prior to the index ED visit, or request by ED clinicians were eligible | n = 2,137  n = 2,406  n = 1,387 | n = 2,137  n = 2,406  n = 1,387  Patients that were not assessed by the ED in reach team. Matched by entropy balancing | Admission to hospital. | NA | Delivery of care | Need for further intervention | Reduced risk of admission (site 1: −9.9% risk of inpatient admission (95% CI = −12.3%, −7.5%), site 2: −16.5% (−18.7%, −14.2%), site 3: −4.7% (−7.5%, −2.0%)). | Readmission | 72 hours   30 days | Resource | Hospital | Increased risk of 72-hour ED revisits for two sites (site 1: 1.5% (0.7%, 2.3%), site 2: 1.4% ( 0.7%, 2.1%)). Risk of any admission within 30 days of the index ED visit remained reduced for sites 1 and 2 (site 1: −7.8% (−10.3%, −5.3%), site 2: −13.8% (−16.1%, −11.6%)). |
| Leung et al [94] | Retrospective cohort Single site 01.09.2015- 31.10.2017  Patients assessed using a frailty assessment pathway compared conventional general care pathway, Data obtained from electronic health record. Matched ( age / sex / vital signs / biochemistry / mobility / Barthel)  Counts compared between groups. | Aged > 65 admitted to a emergency medical ward. Care home patient excluded. | n = 150 | n = 40 | LOS   Readmission | 28 day | Resource use | Hospital | Acute hospital LOS shorter in intervention group (2.38 vs 3.27 days, p = 0.00018). There was no significant difference in the total length of stay (7.10 vs 10.99 days, p = 0.09638) but not difference in total LOS when patents transferred to convalescent hospital included. | NA | NA | NA | NA | NA |
| Marsden et al [95] | Before and after (uncontrolled) Single Site 01.01.2012-31.08.2016  Electronic heath care record and administrative data. Survival analysis was used to jointly model LoS and disposition, with the three destinations (discharged home, admitted to hospital or death) as com- peting risks | Aged > 70 from care homes presenting to ED. | Interim = 3324  Post = 1458 | Pre = 1209 | Admission to hospital | Admission to discharge | Delivery of care | Need for further intervention | HR for discharge for those patients presenting in the post-GEDI intervention period was 1.15 (95% CI 1.05–1.26). | Readmission  LOS  Mortality | 28 days  Admission to discharge  28 days | Resource use    Death | Hospital    All cause hospital mortality | No significant differences |
| Puig et al [96] | Before-after (uncontrolled) Single  01.01-2007.31.12.2017  Used electronic health record to assess outcomes at the hospital level over time. Multifaceted intervention. Increased awareness and training on frailty within the ED | ED attendances and readmission rates over time. Intervention group and comparator groups not reported at the patient level. | Not reported | Not reported | Admission to hospital. | Admission to discharge | Delivery of care | Need for further intervention | Not interpretable. Authors states "It is difficult to establish the program outcomes" | Readmission | 72 hours | Resource | Hospital | Not interpretable. Authors states "It is difficult to establish the program outcomes" |
| Sophia et al [97] | Before and after (uncontrolled) Single site 2-month time period (unspecified)  Described disposition outcomes of those assessed, Compared admission rate with control period in 2012 and national average | Aged >80 presenting to ED with acute medical illness | Not reported | Not reported | Admission to hospital. | Admission to discharge | Delivery of care | Need for further intervention | Reduction in average admission rate compared with a control period prior to implementation. No patient level comparisons | NA | NA | NA | NA | NA |

Table 3D Outcome measures and results

Care home models

| **Author** | **Design** | **Participants** | **Intervention** | **Comparator** | **Primary Outcome** | **Time horizon** | **COMET: core area** | **COMET: domain** | **Result** | **Secondary outcomes** | **Time horizon** | **COMET: core area** | **COMET: domain** | **Result** |
| --- | --- | --- | --- | --- | --- | --- | --- | --- | --- | --- | --- | --- | --- | --- |
| Brickman et al [114] | Prospective cohort Single site  01.04.2016-01.03.2017  Comparison of a single NH with 101 NH in the catchment area of a single ED without access to the model.   Data acquired using electronic health record. Results reported at population level. | All ED presentations amongst NH residents in a single ED | n = 1 (care home)  Number receiving interventions at population level not reported. | n = 101 (care homes) | Unscheduled hospitalisation per 1000 patient days | 1 year | Delivery of care | Admission from ED | 922.9 admissions per 1000 patient days vs 2072.2 in the control group (p < 0.001) | Cost analysis | 1 year | Resource use | Economic | Reduction in lab and radiology costs per patient. |
| Crilly et al [115] | Qualitative evaluation of a nurse-led Hospital in the Nursing Home programme using semi-structured interviews | Semi structured interviews with key stakeholders | n = 19 | NA | NA | NA | Delivery of care | Staff perception | Processes involving strong lines of communication and referral were important for the programme’s successful operation. | NA | NA | NA | NA | NA |
| Crilly et al [116] | Prospective cohort Single site  01.07.2003-30.06.2004  Evaluation of a nurse-led Hospital in the Nursing Home programme. Data acquired using electronic health record | 42 "low-and high-care" NH within the geographic catchment of a single ED. | n = 62 | n = 115 | Total LOS  ED LOS | Admission to discharge | Resource use | Hospital | Significant reduction in LOS in hospital days. 2.1 vs 6.2 days. (p < 0.001)  Intervention group spent longer in ED 9.9 hours vs 7.0 hours (p 0.005) | Readmission | 28 days | Resource use | Hospital | No difference |
| Joseph et al [118] | Retrospective cohort  Single site  01.01.2017-01.01.2018  Telemedicine service provided by an EP. Paramedic or physician associate on site. Data acquired using electronic health record | The control group consisted of residents in NH without intervention transferred to ED. | n = 2311 | n = 2295 | Admission to hospital. Any transfer to hospital from intervention group classed as admission. | Not reported | Delivery of care | Admission from ED | Intervention group less likely to have their care escalated to hospital setting.   27% of patients assessed in intervention group admitted compared with 71% in the control group (OR = 0.15 (95% CI, 0.13-0.17), p < 0.001, | NA | NA | NA | NA | NA |
