# Supplementary material for: Acute care models for older people living with frailty: a systematic review and taxonomy
Source: BMC Geriatr. 2023 Dec 5;23:809. doi: 10.1186/s12877-023-04373-4 (PMC10699071; doi:10.1186/s12877-023-04373-4)
Supplement: Supplementary file 4 — Additional file 4. [file 12877_2023_4373_MOESM4_ESM.docx]

Supplemental table 4

Individual bias assessmentds

Bedded AFU Bias assessments

HaH Bias assessments

ED in-reach bias assessments

Care home bias assessments
